# Supplementary material for: HDAC1 and HDAC2 Modulate TGF-β Signaling during Endothelial-to-Hematopoietic Transition
Source: Stem Cell Reports. 2018 Apr 10;10(4):1369–83. doi: 10.1016/j.stemcr.2018.03.011 (PMC5998800; doi:10.1016/j.stemcr.2018.03.011)
Supplement: Document S1. Supplemental Experimental Procedures and Figures S1–S7 [file mmc1.pdf]

Stem Cell Reports, Volume *10*

Supplemental Information

HDAC1 and HDAC2 Modulate TGF-β Signaling during Endothelial-to--Hematopoietic Transition

Roshana Thambyrajah, Muhammad Z.H. Fadlullah, Martin Proffitt, Rahima Patel, Shaun M. Cowley, Valerie Kouskoff, and Georges Lacaud

Inventory of Supplemental Figures

Supplemental Figure S1: *Hdac1* or *Hdac2* deletion impairs EHT (related to Figure 1 and Figure 2)

Supplemental Figure S2: One allele of *Hdac2* is sufficient for EHT at reduced levels (related to Figure 2 and Figure 3)

Supplemental Figure S3: *Hdac1/2* double knock out diminishes hematopoiesis (related to Figure 3)

Supplemental Figure S4: Apoptosis is increased in *Hdac1/2* (related to Figure 3)

Supplemental Figure S5: Elevated *Caspase3/7* detection in *Hdac1/2* double knock out cultures (related to Figure 3)

Supplemental Figure S6: HDAC1 and HDAC2 ChIP-seq peak distribution (related to Figure 5)

Supplemental Figure S7: Gene expression level of tgfβ, cell cycle and apoptosis related genes (related to Figure 5)

Movie 1 and Movie 2: *Hdac1/2* wild type cultures show low numbers of GFP positive cells (related to Figure 3)

Movie 3- 5: *Hdac1/2* double knock out cells undergo improved EHT with SB43 (related to Figure 6)

A S1: Hdac1 or Hdac2 deletion impairs EHT (related to Figure 1 and Figure 2)

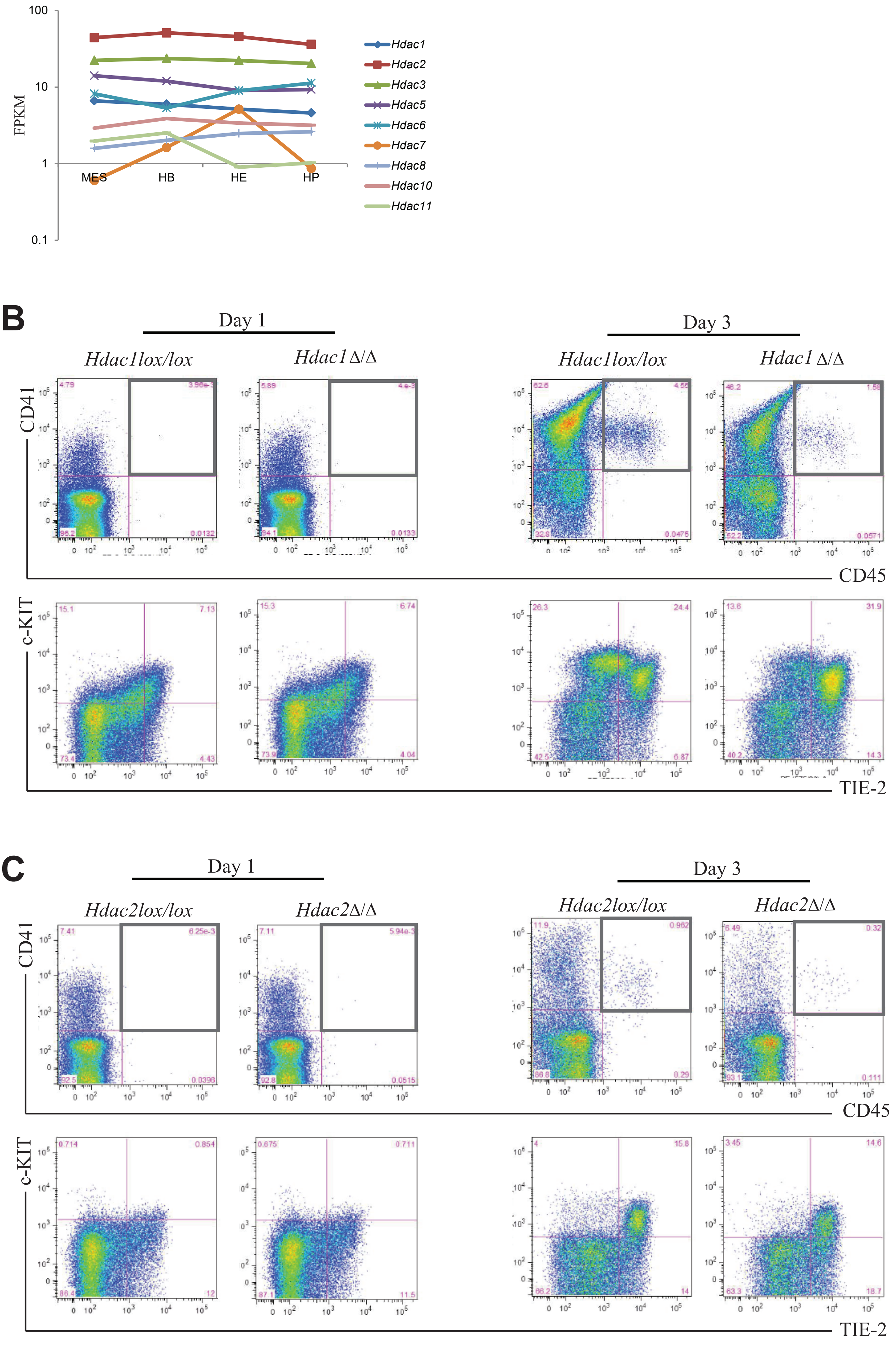

S2 One allele of *Hdac2* is sufficient for reduced EHT (related to Figure 1 and Figure 2)

A

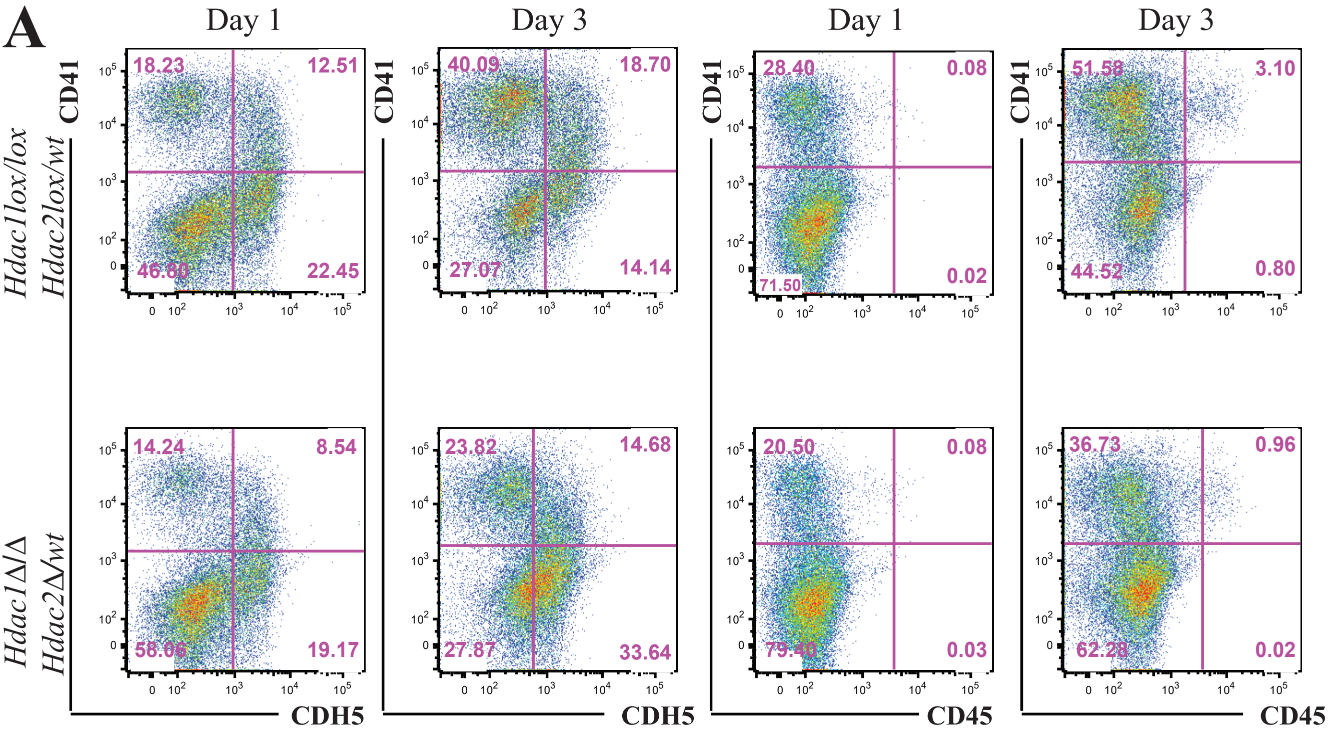

S3: *Hdac1/2* double knock out diminshes hematopoiesis (related to Figure 3)

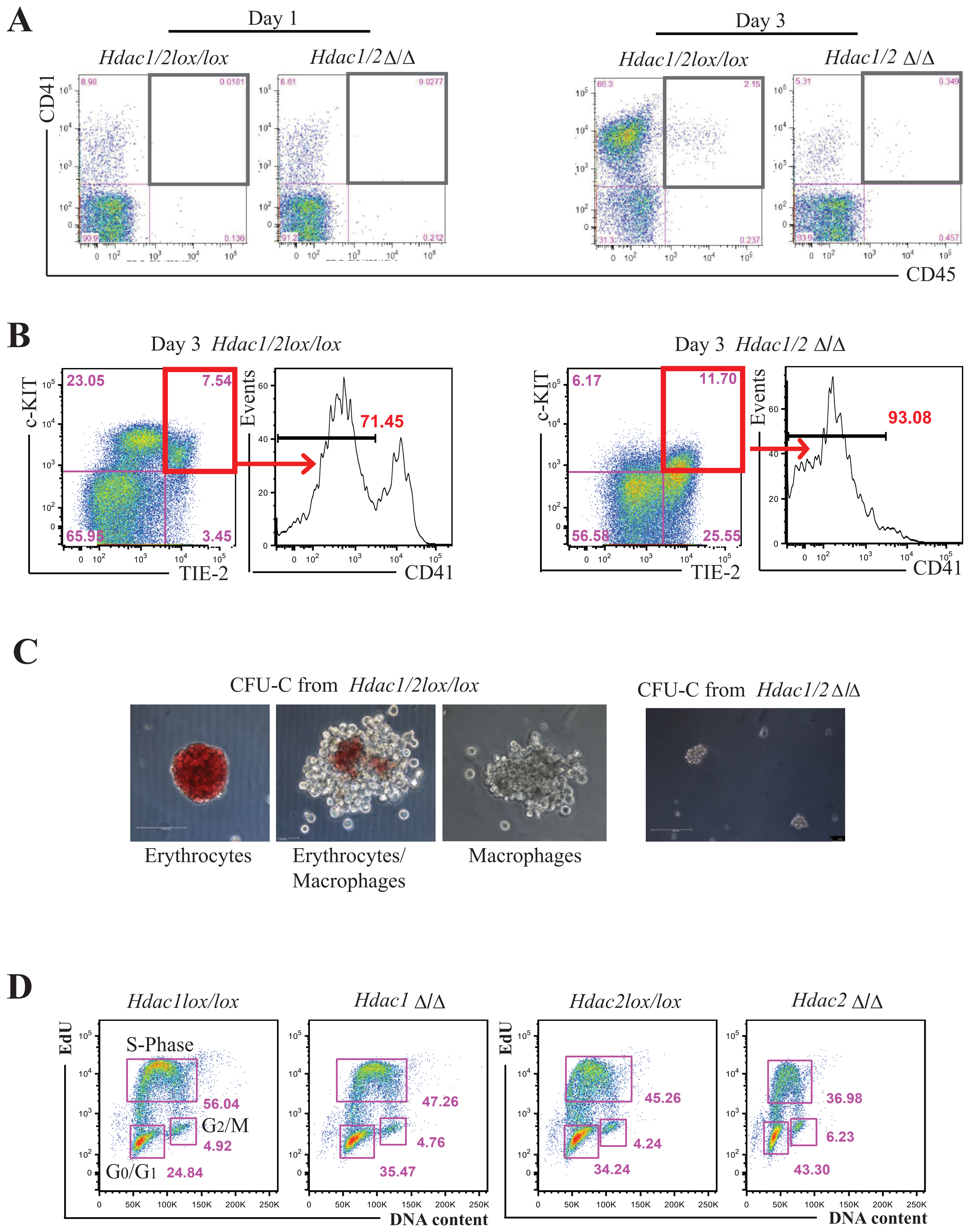

S4 Apoptosis is increased in *Hdac1/2* double KO HE (related to Figure 3)

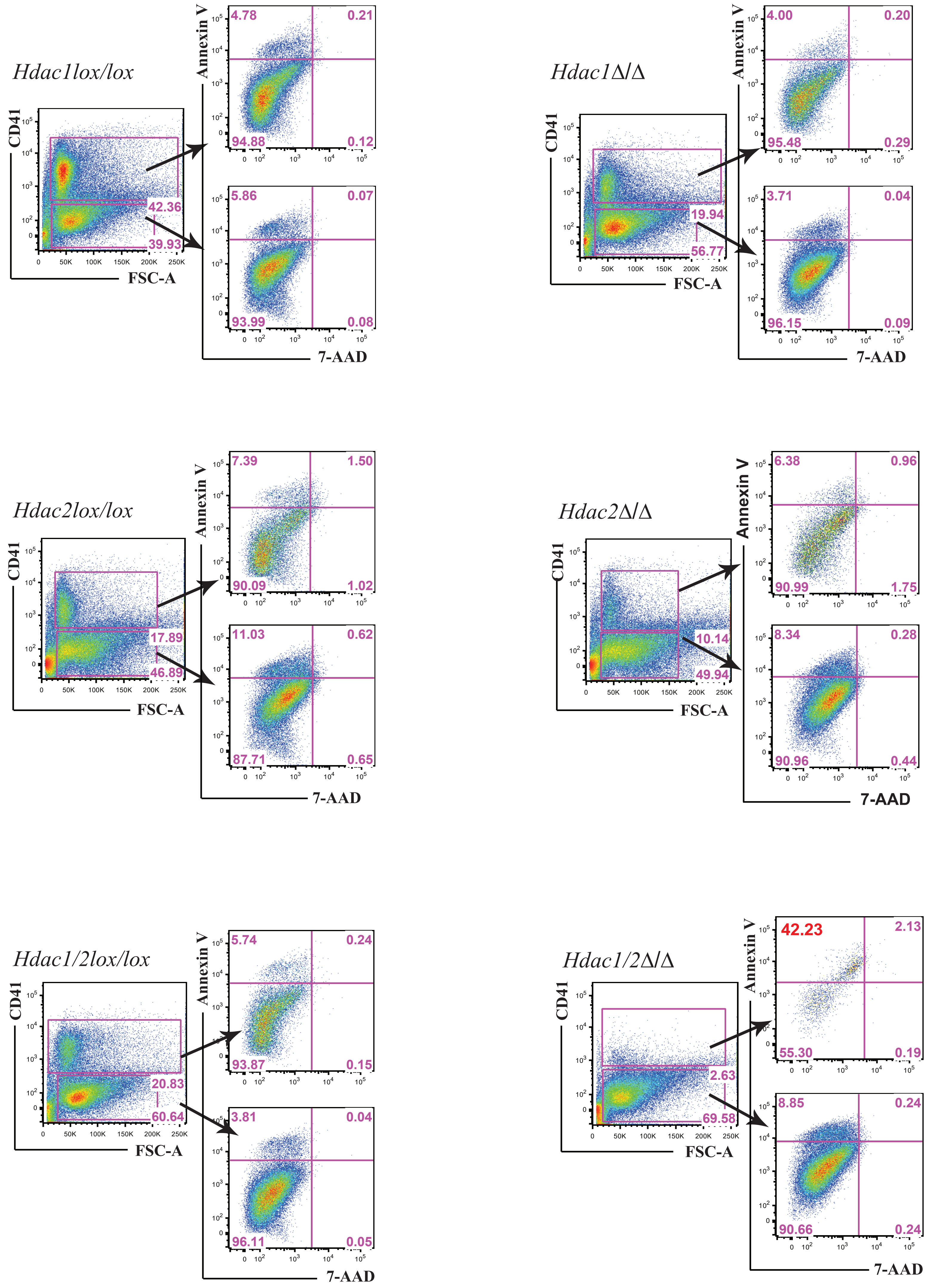

S5 Elevated *Caspase3/7* detection in *Hdac1/2* double KO cultures (related to Figure 3)

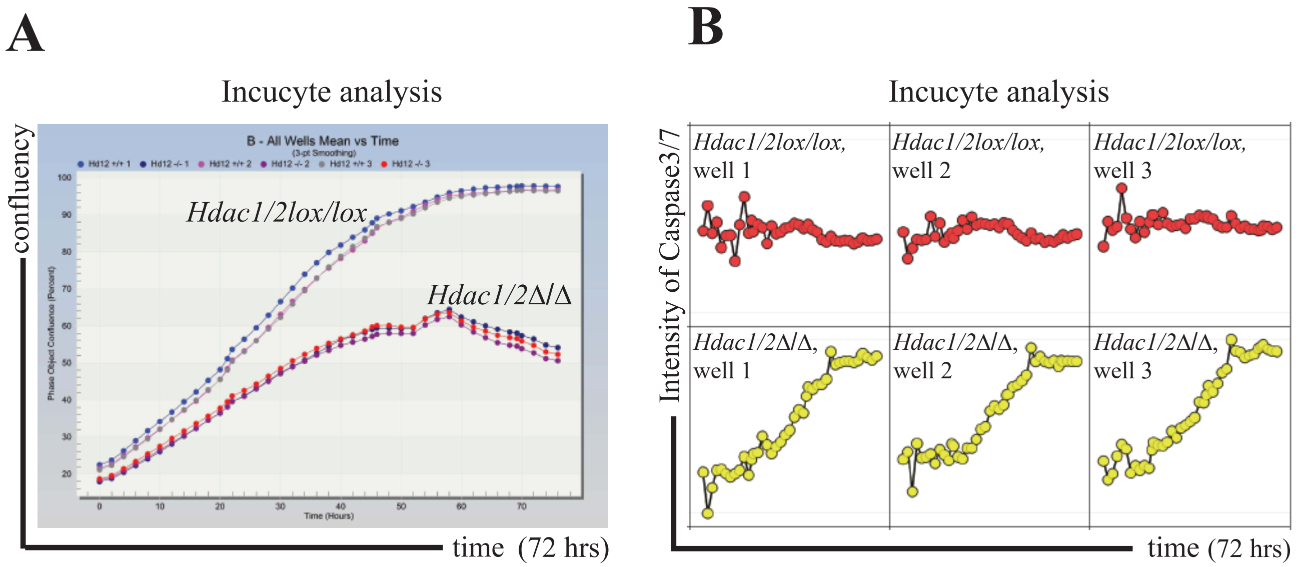

S6: *Hdac1* and *Hdac2* peak distribution (related to Figure 5)

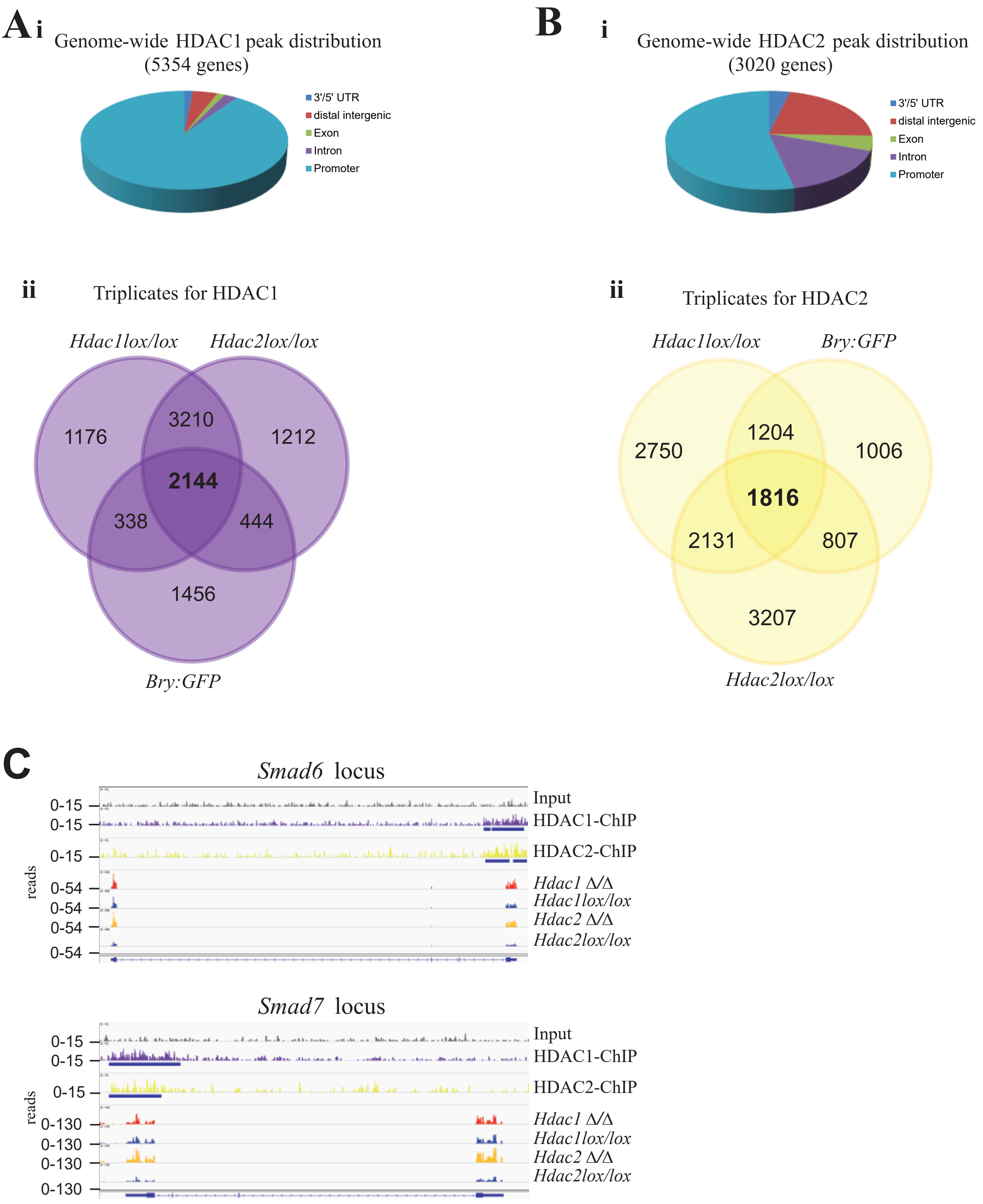

S7 Gene expression level of tgfβ, cell cycle and apoptosis related genes (related to Figure 5)

A Heat map of cell cycle genes

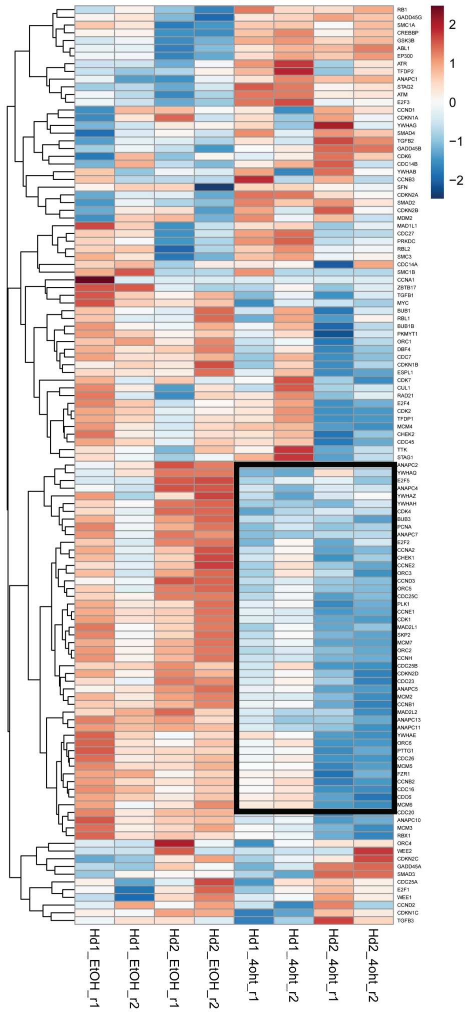

B Heat map of apoptosis genes

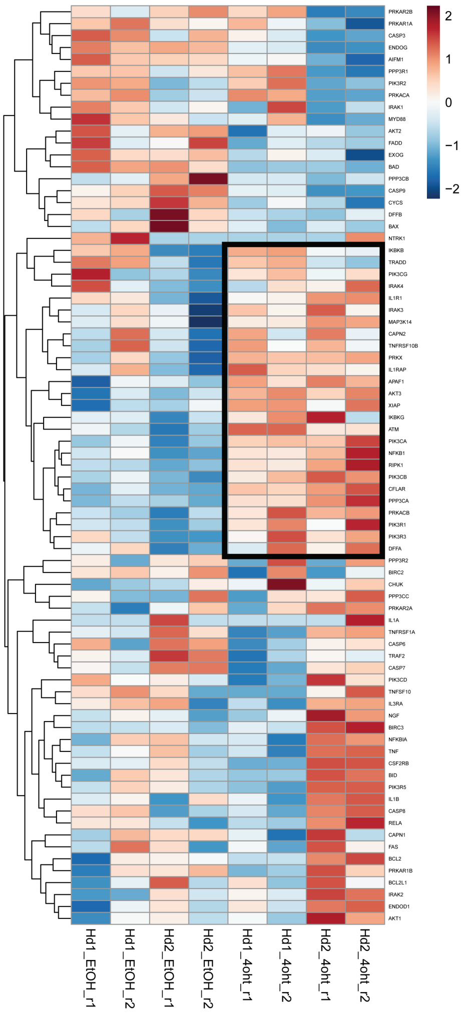

C Heat map of tgfb familiy members

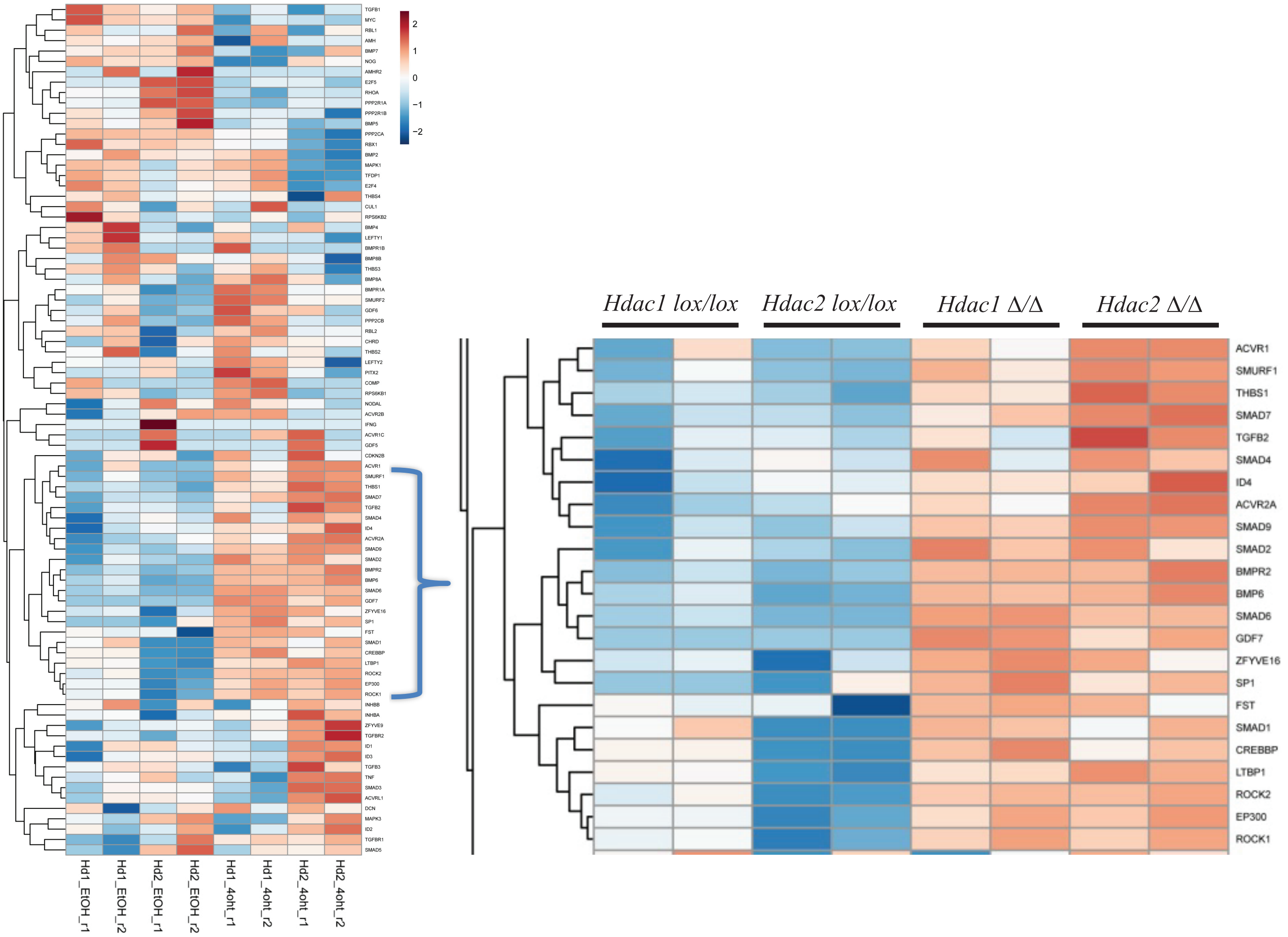

Supplemental Figures

Supplemental Figure S1. HDAC1 or HDAC2 deletion reduces generation of CD45$^+$ cells (A) RNA–sequencing read counts (FPKM= Reads Per Kilobase of transcript per Million mapped reads) for members of the HDAC family in the specified populations. MES= mesoderm, HB= hemangioblast, HE and HP= hematopoietic progenitors (from Goode et al, 2016). (B and C) FACS analysis of *Hdac1loxlox* and *Hdac1Δ/Δ,* or *Hdac2loxlox* and *Hdac2Δ/Δ* cultures at day 1 and day 3. Cells were stained for the endothelial marker CDH5 or TIE2, and the hematopoietic markers c-KIT, CD41 and CD45. Gray boxes indicate CD45 expressing cells shown in Figures 2C and 2F.

Supplemental Figure S2 One allele of *Hdac2* is sufficient for hematopoiesis (A) FACS assessment of day 1 and day 3 *Hdac1loxlox*;*Hdac2lox/wt* and *Hdac1Δ/Δ;Hdac2Δ/wt* cultures for endothelial (CDH5) and hematopoietic (CD41 and CD45) markers.

Supplemental Figure S3 Hematopoietic potential of *Hdac1/2* deleted cells is severely reduced (A) FACS assessment of *Hdac1/2loxlox* and *Hdac1/2Δ/Δ* day 1 and day 3 cultures. Cells were stained for the endothelial marker CDH5 or TIE-2, and the hematopoietic markers c-KIT, CD41 and CD45. Gray boxes indicate CD45 expressing cells shown in Figures 2G. (B) FACS for the endothelial marker TIE-2 and the hematopoietic markers c-KIT and CD41 at day 3 of Li-Blast culture. The red box highlights the cells selected for CD41 expression analysis. (C) Representative images of CFU-C from day 3 *Hdac1/2*lox/lox and *Hdac1/2*Δ/Δ Scale bar: 1mm. (D) FACS plots of EdU cell cycle analysis of day 2 *Hdac1lox/lox*, *Hdac1Δ/Δ*, *Hdac2lox/lox* and *Hdac2Δ/Δ* day 2 Li-Blast cultures. Representative plots of two independent experiments.

Supplemental Figure S4 Apoptosis is significantly increased in *Hdac1/2Δ/Δ* FACS assessment for apoptosis of *Hdac1lox/lox*, *Hdac2*lox/lox and *Hdac1/2*loxlox lines with/out 4OHT treatment. Day 3 cultures were stained for the hematopoietic marker CD41, the cell viability marker 7-AAD and the apoptosis marker Annexin 5. Representative plots of two experiments.

Supplemental Figure S5 Caspase3/7 intensity is significantly increased in *Hdac1/2 Δ/Δ* Extracts from Incucyte time-lapse imaging of the *Hdac1/2loxlox* lines with/out 4OHT, starting from Day1 for 72 hours. (A) Graph showing the confluency of the cultures over 72 hour period. (B) Apoptosis analysis with GFP labelled *Caspase3/7* reagent. GFP is only emitted once it binds to cleaved *Caspase3/7.* The experiment was performed in triplicates.

Supplemental Figure S6 Summary of the ChIP-Seq data for *Hdac1* and *Hdac2* (A) Summary of the ChIP-Seq binding profile for *Hdac1*. (Ai) peak distribution in genomic locations (Aii) Venn diagram of the three ChIP-Seq replicates (B) Summary of the ChIP-Seq data for *Hdac2*. (Bi) peak distribution in genomic locations (Bii) Venn diagram of the three ChIP-Seq replicates. (C) Representative extracts from the Genome Browser (IGV). Two loci, Smad6 and Smad7, are shown with visualisation of the binding sites of HDAC1 and HDAC2 and the RNA-sequencing results of *Hdac1loxlox* and *Hdac1Δ/Δ,* or *Hdac2loxlox* and *Hdac2Δ/Δ*.

Supplemental Figure S7 Heat map of cell cycle, apoptosis and *Tgfβ* related genes Gene lists of interest were retrieved from the GSEA data bank. The expression level of the RNA-Seq data from *Hdac1lox/lox*, *Hdac2lox/lox*, *Hdac1Δ/Δ* and *Hdac2Δ/Δ* was plotted as a heat map for the gene families of interest. (A) Heat map of cell cycle related genes. (B) Heat map of apoptosis related genes. (C) Heat map of *Tgfβ* family members.

Movie 1 and movie 2 *Hdac1/2* wild type cultures show low numbers of GFP positive cells Flk1 positive cells from day 3 EBs of the *Hdac1/2loxlox* line were sorted and treated with ethanol (movie1 *Hdac1/2* wild type) or with 4-OHT in Li-Blast culture (*Hdac1/2* double knock out). One day later, GFP labelled Caspase3/7 antibody was added to the culture and imaged every 2 hours.

Movie 3- 5 *Hdac1/2* double knock out cells undergo improved EHT with SB43 Flk1 positive cells from day 3 EBs of the *Hdac1/2loxlox* line were sorted and treated with ethanol (movie3 *Hdac1/2* wild type), with 4-OHT (*Hdac1/2* double knock out), or 4-OHT and SB43 combined (*Hdac1/2* double knock out with SB43). Starting from a day later, the cells were imaged every hour.

Supplementary Procedures

ES cell culture and in vitro differentiation

For differentiation, ES cells are seeded on irradiated murine embryonic fibroblast feeder cells until 70-80% confluency (2-3 days). After this initial step, the ES cells are primed for differentiation by passaging them twice onto 0.1% gelatin coated plates. During the last passage, the media is switched from DMEM to an IMDM based media. The ES cells are then allowed to differentiate as embryoid bodies (EB) by dissociating ES cells and plating them in low attachment Petri dishes in differentiation media (IMDM,15% FCS, 2 mM l-glutamine (Gibco), 200 μg/ml transferrin (Roche), 0.5 mM ascorbic acid (Sigma) and 4.5×10-4 M MTG) at a density of 25,000-30,000 cells/ml. After 3-3.5 days, hemangioblast progenitors are isolated on the basis of FLK1 expression (Fehling et al., 2003) and replated in hematopoiesis promoting conditions (Li-Blast). For FACS, time-lapse imaging or re-plating into CFU-C assays, these cultures are initiated with 6.5x 10^4 cells per well in a 6-well plate.

CFU-C assay

In brief, 7-10 days old HE/IAHC cultures or day 3 Li-Blast cultures were trypsinized into single cell suspension. For HE/IAHC cultures, 1/10 of the whole culture was plated into three dishes of CFU-C assay, and hematopoietic colonies scored after 7-10 days. For ES cell based Li-Blast cultures, the number of cells in culture was determined, and 25,000 cells were plated in triplicates into CFU-C assay. The hematopoietic colonies were scored 7-10 days later. The CFU-C mix contains the following cytokines and media: Methylcellulose (1.1% final), 1.5 mL of PDS (15% final), PFHM-II (10% final), L -glutamine (2 mM), transferrin (180 mg/mL), MTG (4.5×10^{-4} M), ascorbic acid stock (50 ng/mL), Kit Ligand (KL) (100 ng/mL), IL-3 (1 ng/mL), G-CSF (30 ng/mL), IL-11 (5 ng/mL), erythropoietin (4 U/mL), IL-6 (10 ng/mL), TPO (5 ng/mL), M-CSF (10 ng/mL).

ImmunoHistochemistry

E10.5 embryos were fixed in 4% Paraformaldehyde for two hours, before they were soaked in 30% sucrose and mounted in OCT compound. 10μm sections were prepared using a cryostat. The sections were streptavidin/biotin blocked if a biotin antibody was used followed by serum blocking (PBS with 10% FCS, 0.05% Tween20 and of 10% goat serum (DAKO) for 1 hour before the sections were incubated with primary antibodies at 4°C overnight in blocking buffer. Sections were washed three times in PBST for 15 minutes each and then incubated with fluorochrome-conjugated secondary antibody at room temperature for 1 hour. Sections were further washed three times in PBS and mounted using Prolong Gold anti-fade medium with DAPI (Life

Technologies). Images were taken using a low-light time lapse microscope (Leica) using the Metamorph imaging software and processed using ImageJ.

In vitro culture of AGM cells on OP-9 stromal cells

AGMs of E10-E10.5 embryos were dissected in PBS with 7% fetal calf serum (FCS) and penicillin/streptomycin (100 U/mL). Single cell suspensions were generated by incubating the tissues for 20-30 minutes in 500 µl of 1mg/ml of Collagenase/Dispase (Roche cat. 10269638001) before mechanical dissociation with a syringe and needle. The resulting single cell suspension was used for antibody staining and FACS sorting. Single AGM cells were sorted into individual irradiated (30 Grays) OP-9 coated wells, and cultured for 7 days in re-aggregation medium consisting of IMDM (Invitrogen), 20% fetal calf serum, L-glutamine (4 mM), penicillin/streptomycin (50 units/ml), mercaptoethanol (0.1 mM), IL-3 (100 ng/ml), SCF (100 ng/ml) and Flt3L (100 ng/ml). All growth factors were purchased from Peprotech. Tissues were maintained in 5% CO2 at 37°C in a humidified incubator, and hematopoietic colonies were scored 7-10 days later.

Genotyping PCR

Small pieces of embryonic tissue or yolk sac were dissected off the embryo and placed in PCR tube containing 30ml of PBS. The tissue pieces were boiled for 8 minutes at 98°C for denaturation. The tissues were digested with Proteinase K (50 mg/ml) for 30 minutes at 55°C, and the enzyme deactivated by boiling the samples for a further 10 minutes at 95°C. 1ml of the samples was used as a template for the PCR.

Western Blot

Cells were washed with ice-cold PBS and lysed using NP-40 lysis buffer (150 mM NaCl, 50mM Tris-HCl pH 8, 1% NP-40 and Proteases Inhibitor Cocktail). After 30 min incubation at room temperature, the lysate was centrifuged at 16,000g for 10 min. The supernatants of whole protein extracts were aliquoted and stored at –80°C. Protein concentrations were determined using the Bradford Protein Assay (Bio-Rad). Immediately before loading, aliquots of 30 µg of protein lysates were incubated for 10 min at 70°C in gel-loading buffer (NuPage LDS sample buffer and NuPage reducing agent; Invitrogen). The electrophoresis were performed with Sodium Dodecyl Sulfate Polyacrylamide Gel Electrophoresis (SDS PAGE) with NuPAGE 4-12% Bis-Tris gels in XCell SureLock™ Mini-Cell Electrophoresis System containing MES buffer (Invitrogen). The proteins were transferred onto a nitrocellulose membrane using the iBlot® Gel Transfer Stacks Nitrocellulose (Invitrogen) for 13 min. Immuno blottings were performed with iBind™ Western Device (Thermo Fischer Scientific) according to the manufacturer's protocol. 1:200 dilution of the HDAC1 (Diagenode, pAb-053-050), HDAC2 (abcam, ab16032) and β-Actin (Sigma, A5441) were used. The secondary rabbit and goat antibodies were used at a 1:1000 dilution. Finally, the membranes were developed using the Amersham ECLTM Prime Western Blotting Detection Reagent (GE Healthcare), according to the manufacturer's protocol. Films were exposed and developed in a MAS automated developing machine.

Supplemental References

Dovey, O.M., Foster, C.T., and Cowley, S.M. (2010). Histone deacetylase 1 (HDAC1), but not HDAC2, controls embryonic stem cell differentiation. Proc Natl Acad Sci U S A *107*, 8242-8247.

Fehling, H.J., Lacaud, G., Kubo, A., Kennedy, M., Robertson, S., Keller, G., and Kouskoff, V. (2003). Tracking mesoderm induction and its specification to the hemangioblast during embryonic stem cell differentiation. Development *130*, 4217-4227.

Sroczynska, P., Lancrin, C., Pearson, S., Kouskoff, V., and Lacaud, G. (2009). In vitro differentiation of mouse embryonic stem cells as a model of early hematopoietic development. Methods Mol Biol *538*, 317-334.

Thambyrajah, R., Mazan, M., Patel, R., Moignard, V., Stefanska, M., Marinopoulou, E., Li, Y., Lancrin, C., Clapes, T., Moroy, T.*, et al.* (2016). GFI1 proteins orchestrate the emergence of haematopoietic stem cells through recruitment of LSD1. Nat Cell Biol *18*, 21-32.
