# Supplementary material for: HDAC1 and HDAC2 Modulate TGF-β Signaling during Endothelial-to-Hematopoietic Transition
Source: Stem Cell Reports. 2018 Apr 10;10(4):1369–83. doi: 10.1016/j.stemcr.2018.03.011 (PMC5998800; doi:10.1016/j.stemcr.2018.03.011)
Supplement: Document S4. Article plus Supplemental Information [file mmc9.pdf]

Stem Cell Reports
Article

OPEN ACCESS

HDAC1 and HDAC2 Modulate TGF-β Signaling during Endothelial-to-Hematopoietic Transition

Roshana Thambyrajah,[1] Muhammad Z.H. Fadlullah,[1] Martin Proffitt,[1] Rahima Patel,[1] Shaun M. Cowley,[2] Valerie Kouskoff,[3,*] and Georges Lacaud[1,*]
[1]CRUK Stem Cell Biology Group, CRUK Manchester Institute, 555 Wilmslow Road, Manchester M20 4GJ, UK
[2]Department of Molecular and Cell Biology, University of Leicester, Lancaster Road, Leicester LE1 7RH, UK
[3]Division of Developmental Biology & Medicine, The University of Manchester, Michael Smith Building, Oxford Road, Manchester M13 9PT, UK
*Correspondence: valerie.kouskoff@manchester.ac.uk (V.K.), georges.lacaud@manchester.ac.uk (G.L.)
https://doi.org/10.1016/j.stemcr.2018.03.011

SUMMARY

The first hematopoietic stem and progenitor cells are generated during development from hemogenic endothelium (HE) through trans-differentiation. The molecular mechanisms underlying this endothelial-to-hematopoietic transition (EHT) remain poorly understood. Here, we explored the role of the epigenetic regulators HDAC1 and HDAC2 in the emergence of these first blood cells *in vitro* and *in vivo*. Loss of either of these epigenetic silencers through conditional genetic deletion reduced hematopoietic transition from HE, while combined deletion was incompatible with blood generation. We investigated the molecular basis of HDAC1 and HDAC2 requirement and identified TGF-β signaling as one of the pathways controlled by HDAC1 and HDAC2. Accordingly, we experimentally demonstrated that activation of this pathway in HE cells reinforces hematopoietic development. Altogether, our results establish that HDAC1 and HDAC2 modulate TGF-β signaling and suggest that stimulation of this pathway in HE cells would be beneficial for production of hematopoietic cells for regenerative therapies.

INTRODUCTION

In the adult, hematopoiesis is sustained by hematopoietic stem cells (HSCs) that have the ability to self-renew and to generate all blood lineages. In contrast, during embryogenesis, hematopoiesis is established in successive waves that result in the production of different types of blood lineages (Costa et al., 2012; Medvinsky et al., 2011). The first HSCs emerge intra-embryonically (Cumano et al., 2001; Dieterlen-Lievre, 1975) in the region where the aorta, gonads, and mesonephros (AGM) are localized in the mid-gestation embryo (Medvinsky and Dzierzak, 1996; Muller et al., 1994). Within the AGM, intra-aortic hematopoietic clusters (IAHCs) containing HSCs appear to be associated with the major arteries at embryonic day (E)10.5–E11.5, including the vitelline and umbilical arteries (de Bruijn et al., 2000; Taoudi and Medvinsky, 2007). There, specialized endothelial cells, termed hemogenic endothelium (HE) based on their localization and simultaneous expression of endothelial and hematopoietic markers, trans-differentiate into hematopoietic cells by an endothelial-to-hematopoietic transition (EHT) (Bertrand et al., 2010; Boisset et al., 2010; Kissa and Herbomel, 2010; Taoudi et al., 2008; Zovein et al., 2008). EHT has been shown to promote blood emergence not only in the embryo, but also in the extra-embryonic yolk sac (YS) (Frame et al., 2016) and during *in vitro* differentiation of embryonic stem cells (ESCs) to blood (Eilken et al., 2009; Lancrin et al., 2010; Stefanska et al., 2017).

During ESC *in vitro* differentiation to blood, mesodermal hemangioblasts (HBs), defined as bipotential mesodermal progenitors with endothelial and hematopoietic potential, can be isolated based on FLK1 expression from embryoid bodies (EBs) and instructed to generate blood cells when cultured in hematopoiesis-promoting conditions (Choi et al., 1998; Sroczynska et al., 2009b). During these cultures, VE-cadherin (CDH5)-positive endothelial cells emerge and aggregate as endothelial cores. Within these cores, $CDH5^{+}CD41^{-}$ HE cells, defined as HE1 (Sroczynska et al., 2009a; Stefanska et al., 2017), further progress toward hematopoiesis by acquiring expression of the hematopoietic marker CD41. Spindle shaped $CDH5^{+}CD41^{+}$ HE cells, defined as HE2, then start to round up and bud as hematopoietic cells from the cores. This transition is correlated with concomitant loss of CDH5 expression and gain of CD45 expression by $CDH5^{-}CD41^{+}$ progenitors (Eilken et al., 2009; Lancrin et al., 2009).

The molecular mechanisms underlying the EHT process *in vivo* and *in vitro* remain poorly understood. One of the main drivers of HSC emergence is the transcription factor RUNX1, as its loss leads to a lack of definitive hematopoietic progenitors (HPs) due to a block in EHT (Chen et al., 2009; Lacaud et al., 2002; Lancrin et al., 2009; North et al., 2002; Okuda et al., 1996). Two of its downstream effectors are the transcriptional repressors GFI1 and GFI1B (Lancrin et al., 2012). While loss of either *Gfi1* paralog has no apparent impact on EHT, *Gfi1/1b* double knockout (KO) HE cells cannot undergo EHT (Thambyrajah et al., 2016a, 2016b). *Gfi1*s regulate EHT by recruiting

This is an open access article under the CC BY license (http://creativecommons.org/licenses/by/4.0/).

the CoREST epigenetic remodeling complex in order to induce the silencing of the endothelial identity of the HE (Thambyrajah et al., 2016a). The CoREST complex contains the histone demethylase LSD1, CoREST, as well as both histone deacetylases, HDAC1 and HDAC2. Pharmacological inhibition or genetic deletion of LSD1 impairs the generation of blood cells (Thambyrajah et al., 2016a). These findings suggest that other chromatin-modifying enzymes than LSD1, such as HDACs, could have prominent roles in hematopoietic development. HDAC1 and HDAC2 can function alone or act in higher-order complexes such as the Sin3A, NuRD, and NODE multi-protein repressive complexes, in addition to the CoREST complex (Kelly and Cowley, 2013). As part of these complexes, HDAC1 and HDAC2 proteins modulate gene expression by deacetylating the N-terminal tails of the core histones, resulting in the tightening of the chromatin, which reduces its accessibility for the transcriptional machinery.

The transforming growth factor β (TGF-β) pathway is critical for epithelial-to-mesenchymal transition (EMT) and was also shown recently to be involved in HE formation and EHT (Monteiro et al., 2016). The pathway consists of a phosphorylation cascade; the TGF-β ligands (TGF-β1–4) bind to a type II receptor, which in turn recruits and phosphorylates a type I receptor. Subsequently, the type I receptor phosphorylates receptor-regulated SMADs (R-SMADS) which can now bind the common SMADs (Co-SMADs). R-SMAD/Co-SMAD complexes accumulate in the nucleus where they act as transcription factors to regulate gene expression (Budi et al., 2017). The pathway also includes inhibitory SMADs (I-SMADs) that can block the R-Smad/Co-Smad dimerization. A similar signaling cascade is activated by bone morphogenetic protein (BMP) family receptors and leads to the phosphorylation of SMAD1/5 (pSMAD1/5), whereas active TGF-β/Activin A signaling leads to phosphorylation of SMAD2/3 (pSMAD2/3). The total level of pSMAD2/3 or pSMAD1/5 after stimulation with a ligand are further fine-tuned by a complex auto- and cross-regulations between R-SMADs and I-SMADs (Lebrin et al., 2005).

In this study, we investigated the role of histone deacetylases during EHT first by pharmacologically inhibiting HDAC activity with the pan HDAC inhibitor trichostatin A (TSA). This treatment resulted in a significant impairment in hematopoietic cell generation from ESC-derived HE cells *in vitro* and from AGM HE cells *ex vivo*. We then focused on HDAC1 and HDAC2, which are prevalent members of the HDAC family and implicated in several epigenetic silencing complexes. Our results indicated that deletion of *Hdac1* or *Hdac2* individually resulted in a reduced generation of the $CD41^+$ blood cells from HE. In contrast, the *Hdac1/2* double KO in HE cells led to intact specification toward the endothelial lineage, but cells initiating EHT underwent apoptosis during the process. To define the molecular changes occurring in *Hdac1* and *Hdac2* knockout HE cells, we performed global transcriptomic analysis on these cells, and determined the genome-wide DNA binding patterns of HDAC1 and HDAC2 in the same HE cell population. We found enrichment for members of the BMP and TGF-β signaling pathways among the genes deregulated in *Hdac1*- or *Hdac2*-deficient HE, and bound by the two histone modifiers. We validated the importance of TGF-β modulation by HDACs by partially rescuing the *Hdac1*- or *Hdac2*-deficient phenotype with treatment of the cultures with the TGF-β receptor inhibitor, SB431542 (SB43). Strikingly, addition of SB43 to *Hdac1* and/or *Hdac2* KO cultures did not decrease but increased the frequency of phosphorylated SMAD2/3. Finally, we observed that *ex vivo* treatment with SB43 increases EHT from wild-type AGM and YS HE cells. Altogether, these findings suggest that HDAC1 and HDAC2 activities are critical to modulate the TGF-β signaling pathway and the generation of blood cells through EHT, and that TGF-β activation in HE cells might therefore be beneficial for producing blood cells for regenerative therapies.

RESULTS

HDAC Inhibition Impairs EHT

Having previously shown the critical role of the histone demethylase LSD1 in EHT (Thambyrajah et al., 2016a), we wanted to explore the role of other epigenetic regulators in this process. HDAC proteins were obvious candidates given that they are members of multiple epigenetic silencing complexes. We first tested the impact of the inhibition of HDAC activity on blood formation *in vitro* using the pan-HDAC inhibitor TSA. For this, HBs were isolated from day 3 EBs based on the surface marker FLK1, and then cultured in blood formation-promoting culture conditions (Li-Blast). We treated wild-type cultures with TSA starting either from day 0 (FLK1 stage), day 1 or day 2 of Li-Blast culture, and analyzed the cultures at day 3 by fluorescence-activated cell sorting (FACS) (Figures 1A and 1B). TSA treatment before the onset of EHT, e.g., from FLK1 stage (day 0) or from day 1, dramatically affected the EHT process, which was monitored by the sequential acquisition of CD41 followed by the loss of CDH5 and the gain of CD45 expression. The frequencies of $CD41^+CDH5^-$ cells were severely decreased upon day 0 and day 1 TSA treatments but were not affected when the treatment was initiated from day 2 onward (Figure 1C). However, all the treated cultures presented a decreased frequency of $CD45^+$ cells. These results indicated that inhibition of HDAC activity affects the generation of $CD41^+$ cells, taking

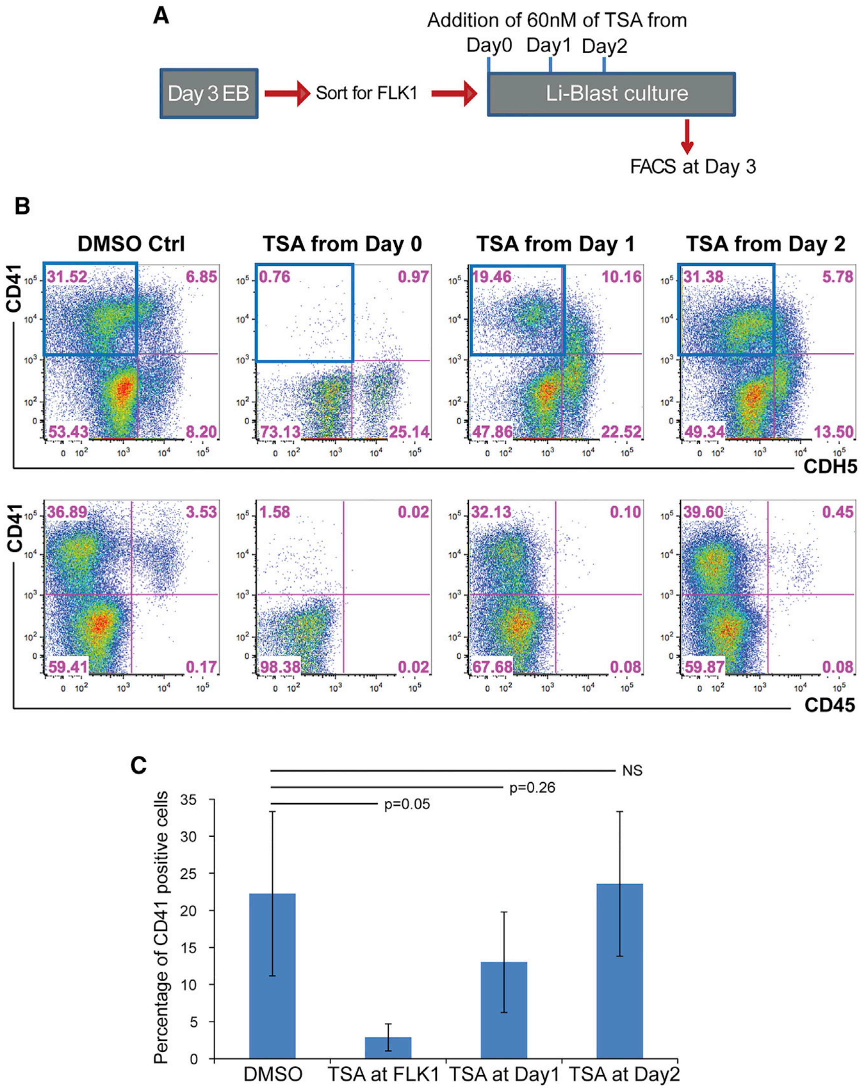

Figure 1. *In Vitro* Differentiation to Blood Is Reduced by HDAC Inhibition

(A and B) Scheme of experimental setup. Flk1^{+} hemangioblasts were isolated from day 3 EBs and plated in Li-Blast cultures. Cultures were treated with 60 nM of TSA at day 0 (FLK1), day 1, or day 2, and (B) assessed by FACS for endothelial (CDH5) and hematopoietic (CD41 and CD45) markers. Blue squares indicate the quadrants depicted in (C). Representative FACS plots from three independent experiments.

(C) Bar chart of averages of CD41^{+} (CDH5^{-}) cells detected in three independent experiments.

Error bars depict SEM. The p values were calculated with two-tailed Student's t test.

place mainly between day 0 and day 2, and the subsequent emergence of CD45^{+} cells. These initial findings suggested a potential requirement for HDAC activity in the EHT.

HDAC1 and HDAC2 were strong candidates for further studies as they are prominent members of the class I HDAC proteins (HDAC1, HDAC2, HDAC3, and HDAC8) that comprise enzymatically active HDACs (Kelly and Cowley, 2013). In addition, our recent study of global gene expression of six sequential stages of hematopoietic specification and differentiation during *in vitro* differentiation to blood (Goode et al., 2016) confirmed that *Hdac1* and *Hdac2* are indeed expressed in HBs, HEs, and HPs (Figure S1A).

Hdac1 and *Hdac2* Are Required for EHT

To circumvent the early developmental defects associated with the loss of HDAC1 or HDAC2 (Montgomery et al., 2007), we employed a conditional KO (cKO) approach to genetically delete them only at the onset of EHT. For this, we made use of *Hdac1lox/lox, Hdac2lox/lox*, and *Hdac1lox/lox/Hdac2lox/lox* ESC lines that constitutively express CRE-ERT2 from the ROSA 26 locus (Dovey et al., 2010; Jamaladdin et al., 2014) allowing induction of CRE and genetic deletion of floxed genes by addition of tamoxifen (4-OHT). To first test the efficiency of *Hdac1* and *Hdac2* deletion, FLK1-positive cells were isolated from *Hdac1* or *Hdac2* cKO EBs and the deletion was induced by activation of the CRE-ERT2 at the FLK1 stage (day 0). Western blots performed on sorted HE cells (CDH5^{+}CD41^{+}) after 2 days of culture indicated a dramatic reduction in HDAC1 or HDAC2 protein levels in the tamoxifen-treated HEs (*Hdac1*Δ/Δ and Hdac2Δ/Δ; Figure 2A). Having validated the system, we focused on the role of HDAC1 and HDAC2 during EHT. Here, we induced the genetic deletions at the FLK1 stage (day 0) and performed FACS analyses at day 1, when the

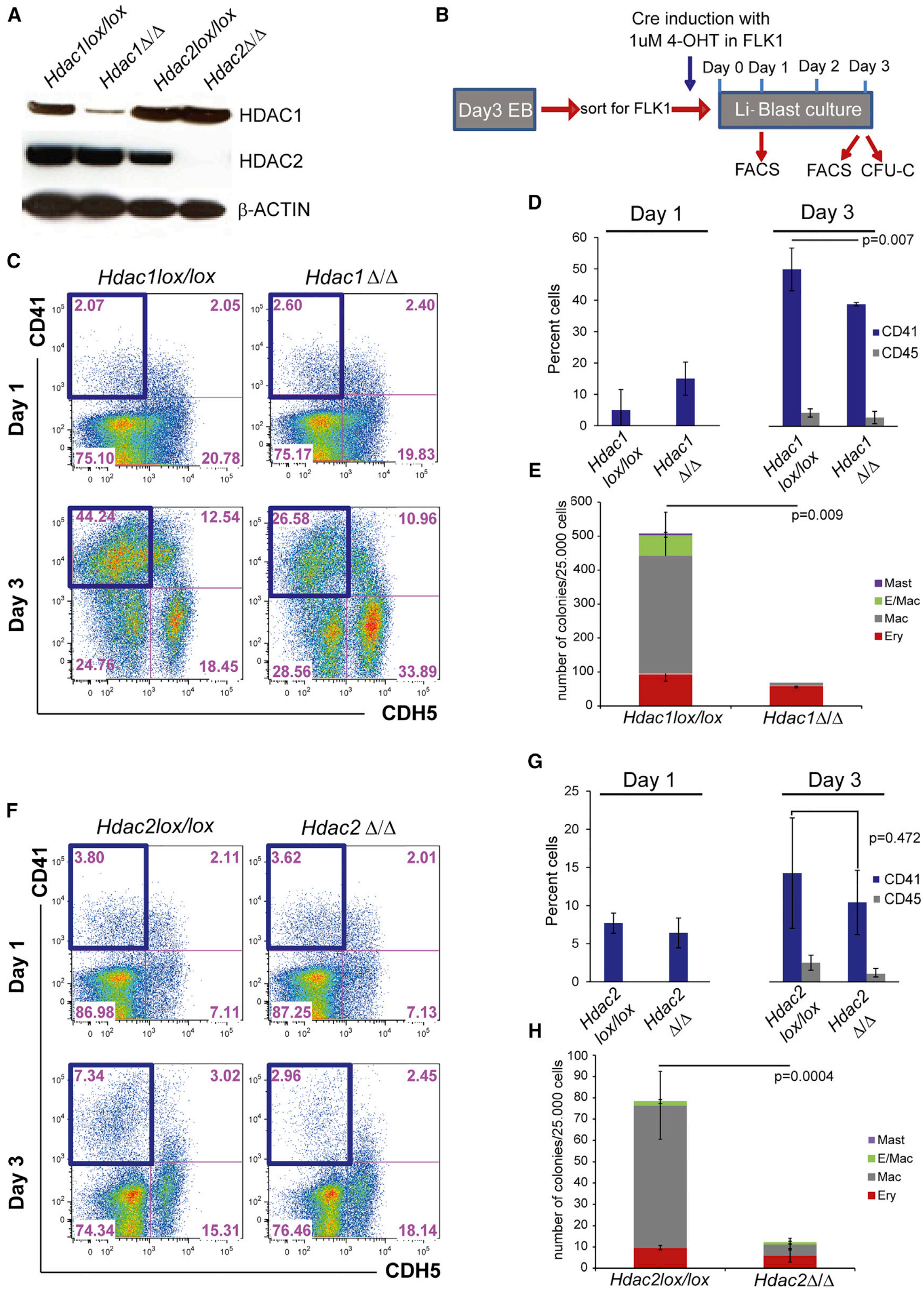

Figure 2. *Hdac1* or *Hdac2* Deletion Reduces Hematopoiesis

(A) Western blot on HE (CDH5$^+$/CD41$^+$) cells derived from control and *Hdac1*- or *Hdac2*-deleted cultures. β-Actin was used as a loading control.

(legend continued on next page)

HE cells started to accumulate. FACS analysis and colony-forming assays (CFU-C) were also performed at day 3, when HPs are present (Figure 2B). We did not observe any significant differences in the frequencies of $CD41^{+}CDH5^{-}$ in *Hdac1*lox/lox or *Hdac1*Δ/Δ by flow cytometry by day 1. In contrast, by day 3, the frequencies of these cells, or the subsequent $CD45^{+}$ cells generated upon successful EHT (acquisition of CD41 followed by loss of CDH5 and gain of CD45 positivity), were significantly lower in *Hdac1*Δ/Δ (Figures 2C, 2D, and S1B). Consistent with an impaired EHT, we observed a marked decrease in the potential of these cells to give rise to different types of hematopoietic colonies (Figure 2E). Similar defects in EHT were observed upon deletion of *Hdac2* (Figures 2F–2H and S1C), suggesting that both HDAC1 and HDAC2 are critical for EHT. In both instances, commitment to an endothelial and HE fate did not seem to be dramatically affected, as indicated by the presence of well-defined cell populations positive for the endothelial marker TIE-2 and the hematopoietic marker c-KIT (Figures S1B and S1C). Actually the requirement for *Hdac1* and *Hdac2* in EHT might be under-evaluated by the time required to deplete the protein levels to a critical threshold, or by redundancy between the two proteins. Indeed, functional compensation between the highly related HDAC1 and HDAC2 has been observed in a number of cell types where deletion of both HDAC proteins led to more severe phenotypes than deletion of a single gene (Dovey et al., 2013; Montgomery et al., 2007; Wilting et al., 2010). To evaluate if this functional redundancy between HDAC1 and HDAC2 took place during EHT, we repeated the experiments, using *Hdac1lox/lox/Hdac2lox/wt* and *Hdac1lox/lox/Hdac2lox/lox* cKO ESCs. Leaving one functional allele of *Hdac2* resulted in reduced frequencies of cells positive for CD41 and CD45 (Figure S2), but mostly recapitulated the phenotype observed following *Hdac1* or *Hdac2* deletion. In contrast, simultaneous deletion of *Hdac1* and *Hdac2* (*Hdac1/2*Δ/Δ) resulted in a dramatic impairment in the generation of $CD41^{+}$ and $CD45^{+}$ hematopoietic cells (Figures 3A, 3B, and S3A). Instead, CDH5-expressing endothelial cells and TIE-2^{+}c-$KIT^{+}CD41^{-}$ HE1 cells accumulated in these cultures (Figures 3A and S3B). Consistent with the severely reduced frequencies of $CD41^{+}$ and $CD45^{+}$ cells, *Hdac1/2* Δ/Δ cultures did not generate any viable hematopoietic colonies in CFU-C assays (Figure 3C) but only very small colonies consisting mostly of dying cells (Figure S3C).

Deletion or knockdown of HDAC protein levels have been previously linked to cell-cycle arrest and increased apoptosis (Wilting et al., 2010). To investigate if impairment in cell-cycle progression was the cause of the severe phenotype observed with the *Hdac1/2*Δ/Δ cells, we performed cell-cycle analysis on day 2 *Hdac1*Δ/Δ, *Hdac2*Δ/Δ, *Hdac1/2*Δ/Δ, and control cultures. The deleted cells displayed only a moderate increase in cells in G_1/G_0 and a limited decrease in the number of cells in the S phase of the cell cycle (Figures 3D and S3D). Moreover, we did not detect any major difference in the cell-cycle status of the double *Hdac1/2*Δ/Δ cells compared with *Hdac1*Δ/Δ or *Hdac2*Δ/Δ single KO cells. In contrast, *Annexin V* staining for apoptotic cells at day 3 revealed a substantial increase in apoptosis, specifically in the $CD41^{+}$ compartment of the *Hdac1/2*Δ/Δ cultures (Figure S4). To further confirm and visualize the presence of apoptotic cells in the double KO, we performed time-lapse imaging on control and *Hdac1/2*Δ/Δ cell cultures in the presence of a reagent that is activated by caspase-3/7 to release a green DNA-binding fluorescent label marking the nuclei of apoptotic cells (IncuCyte Caspase-3/7 Green Apoptosis Assay Reagent). Control cells proliferated exponentially (Figure S5A) and developed to round, floating hematopoietic cells (Figure 3E; Video S1) in the presence of a constant level of Caspase-3/7-positive green cells (Figure S5B; Video S1). In contrast, in the *Hdac1/2*Δ/Δ cultures, the green label

(B) Scheme of the experimental setup. ESCs were differentiated to EBs before isolation of $FLK1^{+}$ cells. Induction of *Hdac1* or *Hdac2* deletion was induced at this stage by addition of 4-hydroxy-tamoxifen (1 μM) in Li-Blast culture. Cultures were analyzed at days 1 and 3 by FACS. At day 3, a fraction of the cultures were also re-plated into CFU-C assays.
(C) Representative FACS analysis at days 1 and 3 of *Hdac1lox/lox* and *Hdac1Δ/Δ* cultures. Cells were stained for the endothelial marker CDH5 and the hematopoietic marker CD41. The blue boxes indicate the $CD41^{+}$ ($CDH5^{-}$) gate used in the bar chart.
(D) Bar chart quantifying the percentage of CD41 ($CDH5^{-}$)- and CD45 ($CDH5^{-}$)-positive cells generated at days 1 and 3 of Li-Blast from *Hdac1lox/lox* and *Hdac1Δ/Δ* cultures. Mean of three independent experiments (n = 3) are shown and p values were calculated with a paired t test.
(E) CFU-C colony assay of day 3 *Hdac1lox/lox* and *Hdac1Δ/Δ* cultures. Mean of three independent experiments (n = 3) are shown with p values calculated with a paired t test.
(F) Representative FACS analysis at days 1 and 3 of Li-Blast of *Hdac2lox/lox* and *Hdac2Δ/Δ* cultures. Staining for the endothelial marker CDH5 and for the hematopoietic marker CD41 is shown. The blue boxes indicate the $CD41^{+}$ gate used in the bar chart.
(G) Mean percentage from three independent experiments (n = 3) of the frequencies of CD41 ($CDH5^{-}$)- and CD45 ($CDH5^{-}$)-expressing cells at days 1 and 3 of *Hdac2lox/lox* and *Hdac2Δ/Δ* cultures are shown. The p values were calculated with a paired t test.
(H) Day 3 *Hdac2lox/lox* and *Hdac2Δ/Δ* Li-Blast cultures were tested for CFU-C activity. Mean colony numbers from three independent experiments (n = 3).
Error bars depict SEM. The p values were calculated with a paired t test.

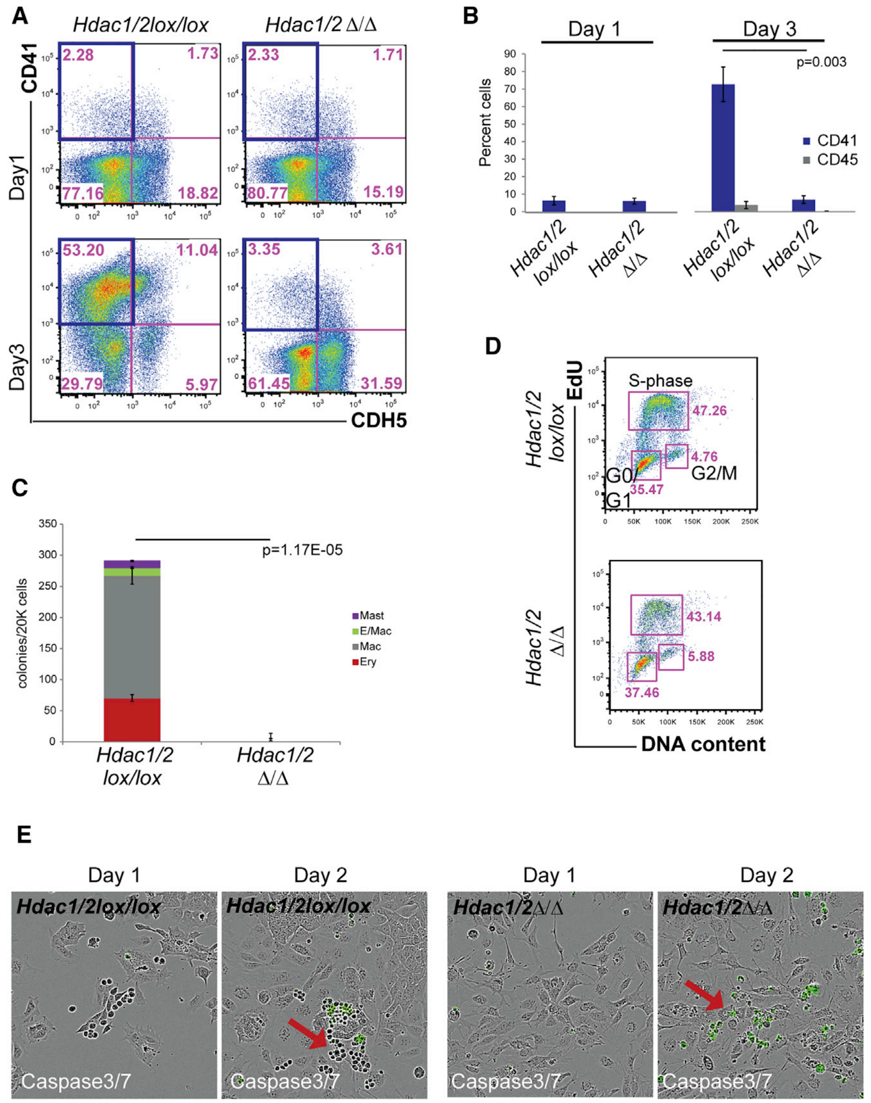

Figure 3. *Hdac1/2* Double Deletion Abolishes Hematopoiesis

(A) Representative FACS analysis of days 1 and 3 of *Hdac1/2lox/lox* and *Hdac1/2Δ/Δ* cultures. Cells were stained for the endothelial marker CDH5 and the hematopoietic marker CD41. The blue boxes indicate the CD41^{+}CDH5^{-} gate used in the bar chart.

(B) Bar graph quantification of the percentage of CD41^{+}CDH5^{-}- and CD45^{+}CDH5^{-}-positive cells detected in day 3 *Hdac1/2lox/lox* and *Hdac1/2Δ/Δ* cultures. Mean of three independent experiments (n = 3). The p values were calculated with a paired t test.

(C) *Hdac1/2lox/lox* and *Hdac1/2Δ/Δ* day 3 cultures were subjected to CFU-C assay. Mean colony numbers from three independent experiments (n = 3) were shown. The p values were calculated with a paired t test.

(D) Representative cell-cycle analysis with 5-ethynyl-2′-deoxyuridine (EdU) on day 3 *Hdac1/2lox/lox* and *Hdac1/2Δ/Δ* cultures. No significant changes were observed between the samples.

(E) Selected images extracted from time-lapse imaging (two independent experiments, n = 2) of *Hdac1/2lox/lox* and *Hdac1/2Δ/Δ* cultures. Cells with activated caspase-3/7 have a green fluorescent nuclear staining, indicative of apoptosis. Arrows depict hematopoietic progenitors.

Error bars depict SEM.

accumulated in adherent cells or in cells rounding up (Video S2; Figure 3E). Altogether these results demonstrate a critical requirement for HDAC1 and HDAC2 in EHT. Loss of either HDAC1 or HDAC2 alone leads to reduced EHT, whereas the combined loss of HDAC1 and HDAC2 proteins results in apoptosis, notably in CD41^{+} cells, corresponding either to adherent HE2 cells or to the subsequent emerging round, hematopoietic cells. The absence of full compensatory redundancy in the single KO also suggests that HDAC1 and HDAC2 have distinct non-overlapping functions.

HDAC Activity Is Critical for *Ex Vivo* Production of Hematopoietic Cells from AGM HEs

We next wanted to evaluate the extent to which HDAC proteins are also required during *in vivo* EHT. We previously established that AGM CDH5^{+}GFI1^{+}c-KIT^{-} and CDH5^{+}GFI1^{+}c-KIT^{+} cells represent, respectively, HE cells and the subsequent cells starting to bud from the endothelial layer to form IAHCs (Thambyrajah et al., 2016a). To evaluate the requirement for HDAC activity in embryonic HE cells during EHT, we isolated these cells from E10.5 AGMs and cultured them on OP-9 stromal cells in the presence or absence of the HDAC inhibitor TSA (Figure 4A). After 7 days, CD41^{+} and CD45^{+} hematopoietic cells were readily detected in the control cultures initiated with either cell population (Figures 4B). In contrast, these hematopoietic cells were largely absent in TSA-treated cultures initiated with either CDH5^{+}GFI1^{+}c-KIT^{-} HE or CDH5^{+}GFI1^{+}c-KIT^{+} IAHC cells (Figure 4B). Consistent with these results, reduced numbers of HPs were generated from CDH5^{+}GFI1^{+}c-KIT^{-} HEs (Figure 4C), and almost no CFU-C were generated from TSA-treated cultures, even

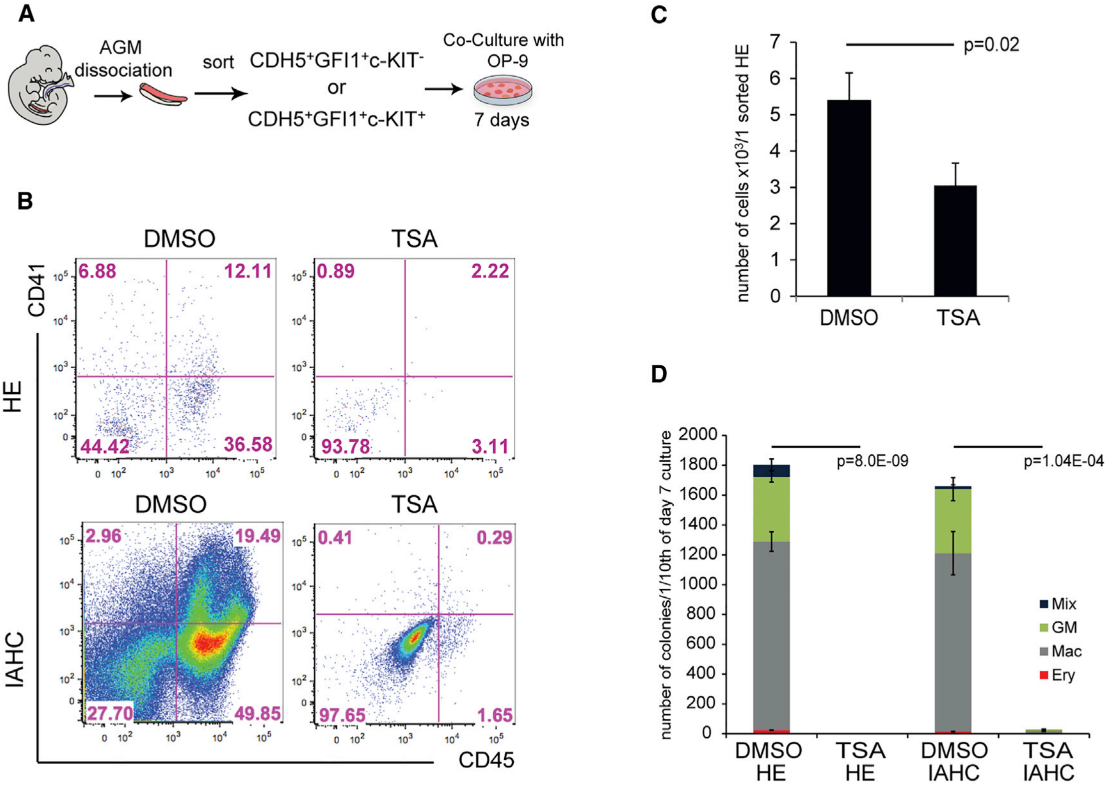

Figure 4. Inhibition of HDAC Activity with TSA *Ex Vivo* Reduces Hematopoiesis

(A) Scheme of the experimental procedure. In brief, E10.5 *Gfi1:tomato* AGMs were dissociated and sorted for HE (CDH5$^+$Gfi1$^+$c-KIT$^-$) and IAHC (CDH5$^+$Gfi1$^+$c-KIT$^+$). The cells were cultured on OP-9 for 7 days and were subjected to FACS and CFU-C assay.

(B) Representative FACS of day 7 HE and IAHC cells cultures with or without 60 nM TSA. Cells were stained for the hematopoietic markers CD41 and CD45.

(C) Hematopoietic output of HE cells calculated per one sorted HE cell from three independent experiments. The p values were calculated with a paired t test.

(D) CFU-C assay performed in triplicates from cultures initiated with 40 cells of HE (n = 2) or IAHC (n = 1) treated or not with 60 nM TSA. Colonies were counted after 7–10 days.

Error bars depict SEM. The p values were calculated with a paired t test.

with the more advanced CDH5$^+$GFI1$^+$c-KIT$^+$ IAHC cells, whereas control cells generated a large number of colonies (Figures 4C and 4D). Overall, these experiments demonstrate a critical role of HDAC activity in the *ex vivo* production of hematopoietic cells from AGM HEs.

HDAC1 and HDAC2 Bind to Endothelial Genes in HEs

To identify the genes directly regulated by HDAC1 and HDAC2, we performed HDAC1 and HDAC2 chromatin immunoprecipitation sequencing (ChIP-seq) experiments on HE2 cells (CDH5$^+$CD41$^+$) from cultures initiated with FLK1$^+$ cells. Analyses of the peaks indicated that HDAC1 binding was mostly found at promoter sites, whereas HDAC2 binding was more heterogeneous, and extended to intronic and intergenic regions (Figures S6Ai and S6Bi). To obtain high-confidence target genes, we performed ChIP-seq with three different ESC lines (*Brachyury:GFP, Hdac1lox/lox*, and *Hdac2lox/lox*) (Figures S6Aii and S6Bii). The resulting datasets were overlapped, and only targets bound in all three replicates were taken forward. These analyses identified 818 genes bound by both HDAC1 and HDAC2 and 1,326 and 998 genes associated with only HDAC1 or HDAC2 binding, respectively (Figure 5A). The gene ontology terms, enriched for the combined binding, showed relevance for cardiovascular system development and artery morphogenesis, supporting a role of HDAC proteins in regulating an endothelial program during EHT (Figure 5A; Table S2), and possibly reflecting in part the role of HDAC proteins in the CoREST complex. We next performed whole genome RNA sequencing on control, *Hdac1ΔΔ*, or *Hdac2ΔΔ* cells (Figure 5B), and found 747 and 3,311 genes differentially expressed between control and *Hdac1ΔΔ* or *Hdac2ΔΔ* cells, respectively. The majority of these genes were upregulated, consistent with

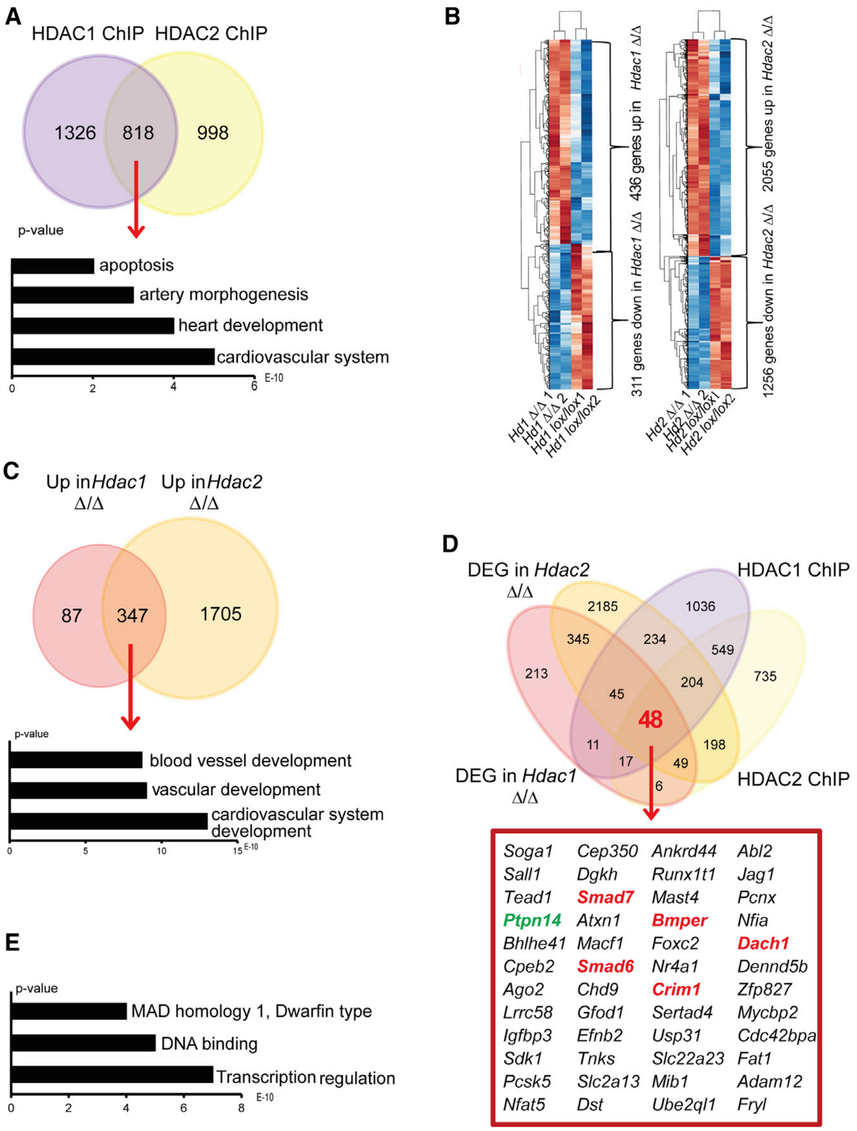

Figure 5. HDAC1 and HDAC2 Regulate Blood Vessel Development and TGF-β Signaling

(A) Venn diagram of the intersection of genes bound by HDAC1 and HDAC2 in HE cells. Gene ontology analysis was performed on the 818 genes bound by HDAC1 and HDAC2. The p value (1.5×10^{-263}) for the overlap probability of the gene lists was calculated with the hypergeometric method.

(B) Heatmap showing the differentially expressed genes in the duplicate samples (n = 2) of *Hdac1lox/lox* versus *Hdac1Δ/Δ* and *Hdac2lox/lox* versus *Hdac2Δ/Δ*. Only genes deregulated more than 1.5-fold are presented.

(C) Venn diagram intersecting the genes upregulated in *Hdac1Δ/Δ* and *Hdac2Δ/Δ*. Gene ontology analysis was performed on the 347 genes upregulated in both KOs. The p value (1.5×10^{-224}) for the probability of the overlap of genes was calculated with the hypergeometric method.

(D) Venn diagram combining deregulated genes in *Hdac1Δ/Δ* and/or *Hdac2Δ/Δ* and bound by HDAC1 and/or HDAC2. Forty-eight genes were present at the intersection of the four groups. Red, negative regulators of *Tgfβ*; green, positive regulator of *Tgfβ*.

(E) Gene ontology analysis on the intersecting 48 genes.

the role of HDAC proteins in transcriptional silencing complexes. In line with our *in vitro* observations, we observed downregulation of cell-cycle-related and upregulation of apoptosis-related genes (Figures S7A and S7B). We also observed deregulation of the expression of members of the TGF-β family (Figure S7C). A total of 347 genes were upregulated upon either *Hdac1* or *Hdac2* deletion, with gene ontology analysis revealing their association with blood vessel, vascular, and cardiovascular system development (Figure 5C; Table S2).

TGF-β Signaling in HEs is Regulated by HDAC1 and HDAC2

We then overlapped the binding data of HDAC1 and HDAC2 with the list of genes that are deregulated in *Hdac1Δ/Δ* or *Hdac2Δ/Δ* HE cells in order to identify candidates that are directly bound and regulated by the HDAC proteins. In total, we were left with 48 genes, which showed enrichment for transcriptional regulation, DNA binding, apoptosis, and MAD homology 1 Dwarfin type related to SMAD proteins (Figures 5D and 5E). Indeed, several members and regulators of the TGF-β pathways were present, including PTPN14, SMAD7, SMAD6, CRIM1, BMPER, and DACH1 and the expression of all these genes was upregulated in *Hdac*-deleted HEs (Figures 5D and S6C; Table S2). To validate our findings, we queried our recent survey from a previous study of global gene expression during six sequential stages of hematopoietic specification and differentiation (Goode et al., 2016). Most of these members of TGF-β pathways were expressed at the

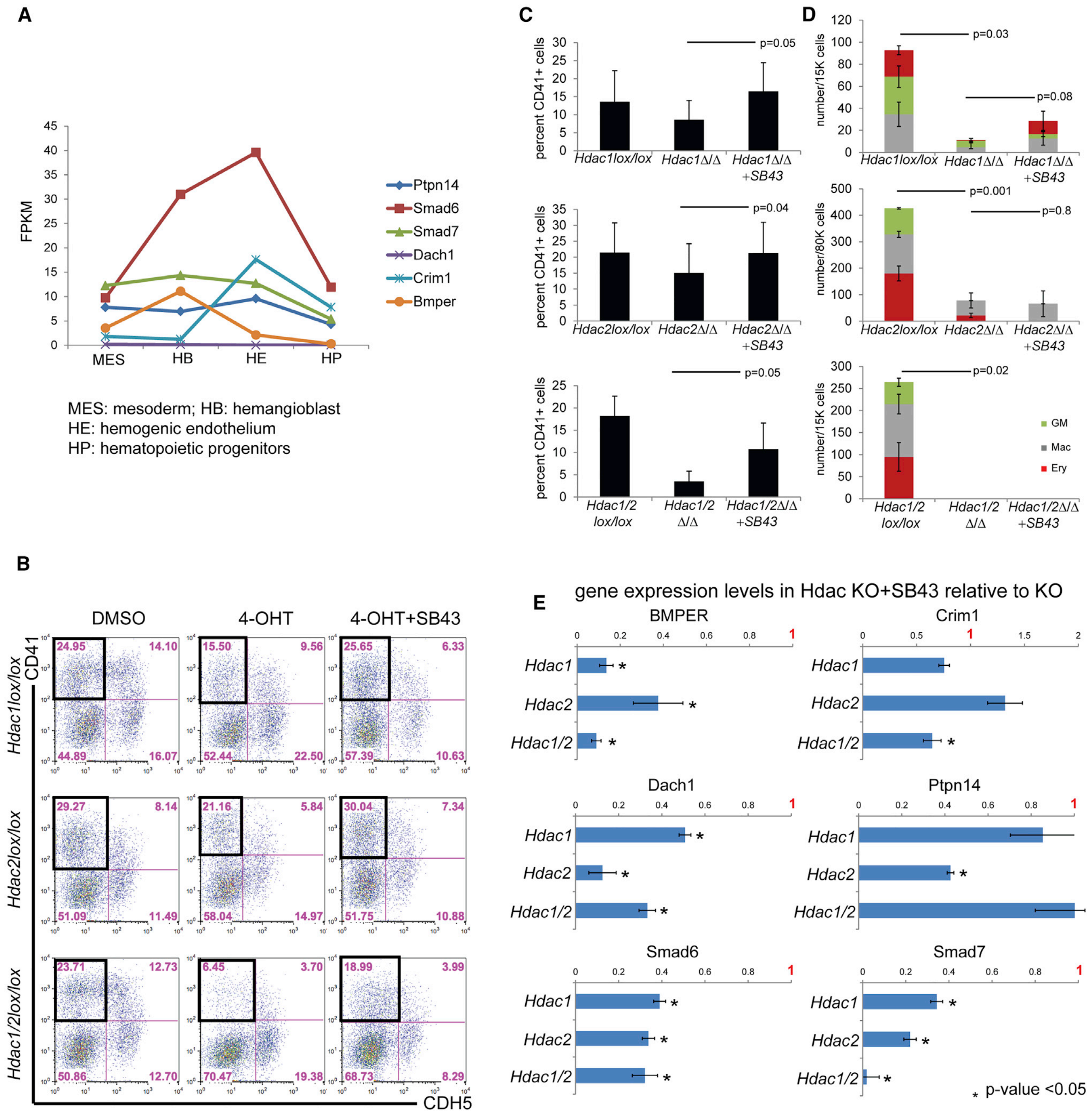

Figure 6. SB43 Treatment Increases Hematopoiesis

(A) RNA sequencing read counts (FPKM, reads per kilobase of transcript per million mapped reads) for the genes of interest in specified populations; MES, mesoderm; HB, hemangioblast; HE, hemogenic endothelium; and HP, hematopoietic progenitors.

(B) *Hdac1lox/lox* and *Hdac2lox/lox* and *Hdac1/2lox/lox* treated with 4OHT or 4OHT + SB43. Day 3 cultures were stained for the endothelial marker CDH5 and the hematopoietic markers CD41. The black box indicates the $CD41^{+}CDH5^{-}$ gate used for the bar chart in (C).

(C) Bar chart of averaged $CD41^{+}CDH5^{-}$ cells in DMSO control (*Hdac1* and/or *Hdac2* wild-type), 4OHT (*Hdac1* and/or *Hdac2* deletion), or 4OHT + SB43 (*Hdac1* and/or *Hdac2* deletion with SB43) from three independent experiments. The p values were determined with a paired t test.

(D) Clonogenic assay (CFU-C) of DMSO ctrl (*Hdac1* and/or *Hdac2* wild-type), 4OHT (*Hdac1* and/or *Hdac2* deletion), or 4OHT + SB43 (*Hdac1* and/or *Hdac2* deletion with SB43) from three independent experiments. The p values were determined with a paired t test.

(legend continued on next page)

HB and HE stage, and their expression decreased from the HE to the HP stage (Figure 6A). These results suggest that modulation of the TGF-β pathway through HDAC activity might be an important step in EHT.

TGF-β Activation Promotes EHT

We next performed a series of experiments to test the influence of TGF-β signaling during *in vitro* differentiation of the *Hdac1* or *Hdac2* ESC lines, by adding either TGF-β1 to activate the TGF-β pathway or, alternatively treated *Hdac* KO cultures with SB431542 (SB43), a selective inhibitor of the receptor ALK5, and its relatives ALK4 and ALK7. Treating the cultures with TGF-β1 did not significantly alter the generation of $CD41^+$ cells as observed by day 3 in cultures of *Hdac1*lox/lox*, Hdac2* lox/lox, *Hdac1*Δ/Δ, or *Hdac1/2*Δ/Δ (data not shown). In contrast, treatment with SB43 led to a restoration of normal frequencies of $CD41^+CDH5^-$ cells generated, as assessed by FACS in all the *Hdac* KO cultures (Figures 6B and 6C). Accordingly, time-lapse imaging of *Hdac1/2*Δ/Δ indicated that addition of SB43 rescued the budding of round cells, although these cells were not able to expand further (Videos S3–S5). Similarly, when the cells were assayed in the CFU-C assay, we observed a limited rescue of the blood colony-forming abilities only for the *Hdac1* KO (Figure 6D). These data suggest that modulation of TGF-β signaling is sufficient for partially rescuing the impaired EHT in the absence of HDAC1 and/or HDAC2, but not for restoring further proliferation and survival. Finally, qPCR analysis on isolated HEs of the *Hdac1*, *Hdac2*, and *Hdac1/2* KO HEs treated with SB43 indicated significant reduction in the gene expression level of the TGF-β members that were upregulated in the *Hdac* KO cultures (Figure 6E), consistent with the hypothesis that the observed EHT defects in *Hdac1*, *Hdac2*, and *Hdac1/2* KO cultures were indeed caused by a deregulation of TGF-β. To further confirm these findings, we determined the overall pSMAD2/3 levels in *Hdac1*, *Hdac2*, and *Hdac1/2* KO HE with and without SB43 treatment, as a readout for TGF-β signaling. We performed imaging using flow cytometry for the HE markers CDH5 and CD41 combined with intracellular detection of pSMAD2/3 and DAPI. In line with the elevated levels of negative regulators of TGF-β (SMAD7, SMAD6, CRIM1, BMPER, and DACH1) in *Hdac1*, *Hdac2*, and *Hdac1/2* KO HEs, we observed significant reduction of total pSMAD2/3 in the *Hdac* KO HEs compared with the non-deleted counterpart (Figures 7A and 7B). More surprisingly, pSMAD2/3 levels in the *Hdac1*, *Hdac2*, and *Hdac1/2* HEs was restored to wild-type levels upon SB43 treatment (Figures 7A and 7B), thereby indicating that the rescued EHT is associated with restoration of TGF-β signaling. To further validate our findings in the context of *in vivo* EHT, we performed immunohistochemistry for pSMAD2/3 in combination with staining for the endothelial marker CD31, and the IAHC marker CD41, on E10.5 AGM sections. We detected pSMAD2/3 accumulation in budding cells and hematopoietic clusters of the AGM (Figures 7C and S8), further correlating a successful EHT with activation of TGF-β signaling. Finally, we examined if modulation of TGF-β signaling could also be implicated in EHT by AGM or YS HE cells. For this, we sorted individual single $CDH5^+GFI1^+c\text{-}KIT^-$ HE cells into single wells in the presence of OP-9 stromal cells (Figure 7D). The cultures were treated with either DMSO (control) or SB43. Assessment of the hematopoietic potential after 7 days of culture revealed a significant increase in the frequency of blood colonies in SB43-treated cultures. We also observed significantly larger colonies in these cultures compared with control-treated cultures (Figure 7E). Therefore SB43 treatment not only increases EHT frequency, but also has a proliferative effect on the emerging progenitors. Altogether these experiments indicate that TGF-β activation promotes EHT, and that the defective EHT observed in Hdac1Δ/Δ, Hdac2Δ/Δ, and Hdac1/2Δ/Δ, cultures could be, to some extent, rescued by activation of TGF-β signaling.

DISCUSSION

The molecular and cellular regulation of blood cell formation through EHT remains poorly understood. We previously determined that the CoREST epigenetic silencing complex, and in particular the histone demethylase LSD1, is implicated in the downregulation of the endothelial program during the transition from endothelial to hematopoietic cells (Thambyrajah et al., 2016a). However, the potential functions of the other chromatin-modifying proteins, such as HDACs, in the EHT process are just starting to be explored. Indeed, several HDACs were shown to be implicated in the generation of definitive blood formation, notably through reverse genetic screens in zebrafish (Burns et al., 2009; Huang et al., 2013), but the exact stages of blood cell development affected remain poorly characterized. In one of these studies, *Hdac1* was shown to be required for *Runx1* expression downstream of *Notch* signaling, suggesting an early role before EHT. In a follow-up study, HDAC1, HDAC2, and HDAC9a were

(E) qPCR analysis of the six *Tgfβ*-related candidates on sorted HE cells. The expression levels of the indicated gene was normalized to β-actin and the value obtained in the Hdac1 and/or Hdac2 KO HE without SB43 set to a value 1. The value upon SB43 treatment was plotted relative to this level of 1 obtained without SB43. Averages from two independent experiments (n = 2).
Error bars depict SEM.

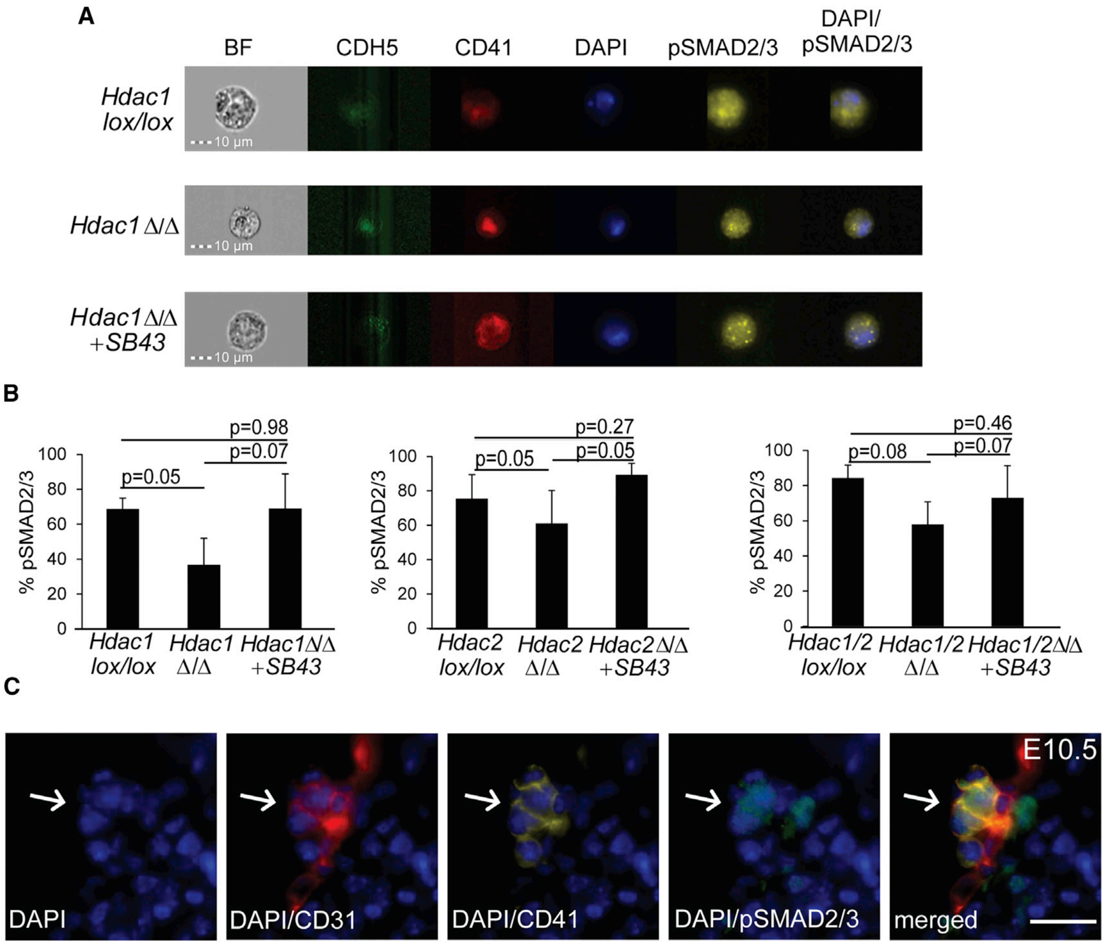

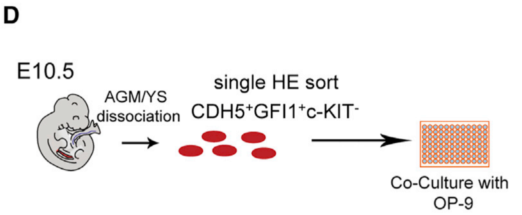

	exp #1 (AGM)	exp #2 (AGM)	exp #3 (AGM)	exp #4 (AGM)
DMSO	2/32	2/32	0/32	2/32
SB43	5/32	3/32	3/32	3/32

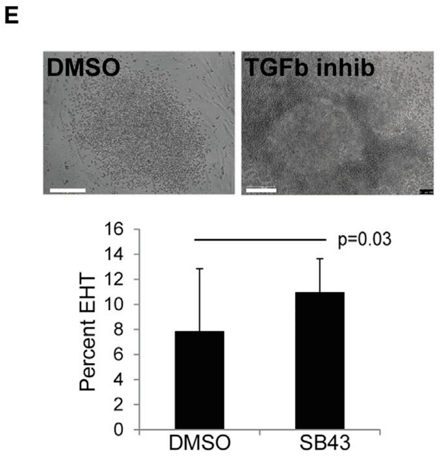

Figure 7. SB43 Treatment Increases TGF-β Signaling in *Hdac1* and/or *Hdac2* KO HE

(A) Representative pSMAD2/3 levels in *Hdac1lox/lox*, *Hdac1Δ/Δ*, and *Hdac1Δ/Δ*+SB43 from ImageStream analysis.

(B) Averaged pSMAD2/3 levels from three independent experiments of the indicated cultures. The p values were calculated with a paired t test.

(C) Immunohistochemistry for the endothelial marker CD31, IAHC marker CD41, and pSMAD2/3 levels on E10.5 AGM section. Arrows depicts IAHC. Scale bar, 10 μm.

(legend continued on next page)

proposed to function, notably through an HDAC-NuRD deacetylase complex, in the specification or maintenance of hematopoietic cells from the HEs. This would therefore be compatible with a role of these HDACs in EHT.

In this study, we pharmacologically inhibited HDAC activity with the pan-HDAC inhibitor TSA and observed a significant impairment in the generation of hematopoietic cells from ESC-derived HE cells *in vitro* and from AGM HE cells *ex vivo*. We then focused on HDAC1 and HDAC2, which are implicated in several epigenetic silencing complexes, and therefore could be implicated in the EHT as part of one or several of these complexes. Accordingly, we demonstrated a significantly reduced EHT upon HDAC1 and/or HDAC2 deletion. Consistent with functional redundancy and compensation between HDAC1 and HDAC2, we found a much more severe block in EHT in the absence of both proteins. Loss of both HDAC proteins led to a dramatic increase in apoptosis, specifically in $CD41^+$ cells that are undergoing EHT. Interestingly, the deletion of Sin3a has also been shown to be associated with increased apoptosis (Cowley et al., 2005; Dannenberg et al., 2005), suggesting that the role of HDAC1 and HDAC2 identified here could reflect their function as part of the Sin3a repressive complex. We also found that HDAC1 and/or HDAC2 genetic deletions and binding was associated with genes implicated in cardiovascular system development. These findings would be compatible with roles of HDAC1 and HDAC2 in the CoREST repressive complex previously shown to be critical for the downregulation of the endothelial program during EHT.

In addition, our results suggested that HDAC1 and HDAC2 regulate hematopoietic emergence through the modulation of the TGF-β signaling pathway. Accordingly, we experimentally demonstrated both *in vitro* and *in vivo* that modulation of this pathway in HE cells reinforces hematopoietic development and that activation of TGF-β signaling is associated with a successful EHT. TGF-β is strongly associated with EMT that, like EHT, requires loss of cell-cell contact and change in morphology. EMT and its reverse process, mesenchymal-to-epithelial transition, are naturally occurring processes during development that are hijacked in cancers (Thiery et al., 2009). The main hallmarks of EMT are induction of the TGF-β downstream effectors *snail/slug* and *zeb1/2* genes (Thiery et al., 2009). The implication of TGF-β in the EHT is further supported by recent RNA sequencing of AGM populations, including HE and IAHC, which revealed *snail* and *zeb* expression during the EC to pre-HSC transition (Zhou et al., 2016) and their specific accumulation in the hematopoietic competent part of the dorsal aorta (McGarvey et al., 2017). Similarly, in agreement with an essential positive requirement for TGF-β in the EHT, knockdown of the receptor TGF-βRII, or the ligands TGF-β1 or TGF-β, were shown to impair HE specification (Monteiro et al., 2016). In contrast, a recent study suggested that activation of TGF-β signaling impairs EHT (Vargel et al., 2016). However, this was mostly based on results obtained with SB43, which was considered as a negative regulator of the pathway, whereas in our hands SB43 surprisingly leads to increased pSMAD2/3 signaling. Our observations were confirmed by recent findings showing that SB43 only has a negative impact on pSMAD2/3 levels for a very short time. After this initial phase of repression, SB43 induces an increase in pSMAD2/3 levels by an unknown mechanism (Ruetz, 2017). Interestingly, these authors argue for a role of pSMAD2/3 in cell fate switching, and acceleration of this switch, which would be compatible with our findings. Indeed, we detected an increase in E10.5 AGM HEs undergoing EHT following SB43 addition, suggesting that the HE population has more EHT competent precursors than detected in the wild-type setting. Overall, TGF-β signaling in HE cells could therefore participate in their cell fate conversion to hematopoietic cells.

In conclusion, we have established here that HDAC1 and HDAC2 are individually required but not essential for hematopoietic emergence. In contrast, deletion of both genes results in apoptosis of $CD41^+$ cells undergoing EHT and a complete lack of functional HP generation. We also demonstrated that HDAC1 and HDAC2 regulate EHT at least in part through the modulation of the TGF-β signaling pathway. These results suggest that modulation of HDAC activity and TGF-β activation in HE cells might be beneficial for producing blood cells through *in vitro* differentiation or reprogramming.

EXPERIMENTAL PROCEDURES

ESC Line Growth and Differentiation

Bry-GFP (Fehling et al., 2003), *Hdac1lox/lox*, *Hdac2lox/lox Hdac1lox/loxHdawt/lox*, and *Hdac1/2lox/lox* ESC lines (Dovey et al., 2010) were used. ESC cultures, maintenance, and differentiation and composition of the different media were described previously (Sroczynska et al., 2009a, 2009b). For SB431542 treatment,

(D) Hematopoietic colony assay on single cells. E10.5 *Gfi1:tomato* AGMs were dissociated and sorted for HE ($CDH5^+Gfi1^+c\text{-}KIT^-$) and cultured on OP-9 for 7 days.
(E) Single HE cells ($CDH5^+Gfi1^+c\text{-}KIT^-$) from E10.5 AGM (n = 4) were sorted into individual wells with or without SB43. Colonies were scored and images were taken 7 days later. Scale bar, 100 μm.
Error bars depict SEM.

10 μM of the compound was added to the Li-Blast culture at FLK1 stage.

Time-Lapse Imaging of Li-Blast Cultures and Caspase-3/7 Staining Assay

*Hdac1/2*lox/lox ESC lines were cultured and differentiated as described above. For time-lapse imaging, day 3 FLK1^{+} cells were replated at 6.5×10^4 cells per well in six-well plates and time-lapse imaging was initiated the following day using an IncuCyte Zoom device (Essen Instruments). For caspase-3/7 staining assay, a 1:200 dilution of the caspase-3/7 GFP reagent was added to stain apoptotic cells (IncuCyte Caspase-3/7 Green Apoptosis Assay Reagent, cat. no. 4440). The movies were processed and labeled with Final Cut Pro X (Apple).

CFU-C Assays

CFU-C assays were performed as described previously (Sroczynska et al., 2009b).

Mouse Lines and Animal Work

The *Gfi1:tomato* mouse line have been described previously (Thambyrajah et al., 2016a). For time matings, wild-type ICR females (aged 6–12 weeks) were mated to *Gfi1^{tomat}* males (C57/Bl6, aged 6–20 weeks). These embryos were genotyped and used for AGM/YS HE or IAHC sorts. Vaginal plug detection was considered as day 0.5. All animal work was performed under regulation in accordance with the United Kingdom Animal Scientific Procedures Act (ASPA) 1986 and was approved by the Animal Welfare and Ethics Review Body (AWERB) of the Cancer Research UK Manchester Institute.

In Vitro Culture of AGM Cells on OP-9 Stromal Cells

In vitro culture of AGM cells was performed as described previously (Thambyrajah et al., 2016a).

EdU and Annexin V Staining

5-Ethynyl-2'-deoxyuridine (EdU) and Annexin V/7AAD staining were performed on days 2 and 3 of blast cultures according to the manufacturer's instructions (Click-iT EdU Alexa 647, Invitrogen, cat. no. C10419 and AnnexinV Apoptosis Detection Kit from eBioscience, cat. no. 88-8007-72) and analyzed on an LSRII (BD Biosciences).

pSMAD2/3 Stain for ImageStream Analysis

Day 2 Li-Blast cells were fixed for 15 min at room temperature in 4% paraformaldehyde and subsequently permeabilized in 90% methanol overnight at −20°C. Cells were stained according to the manufacturer's instructions. The samples were washed and run on Amnis Imaging Flow Cytometer X. Data were analyzed with IDEAS software.

Flow Cytometry and Cell Sorting

Stained cells were analyzed on a Fortessa (BD Biosciences). Sorts were performed on FACSAria III, BD Influx (BD Biosciences), or by magnetic sorting (Miltenyi Biotec). The antibodies and streptavidin used were FLK-1-bio, TIE2-PE, c-KIT-APC-eFluor780, CD41-PE, CDH5–APC, CD41-PE-Cy7, or CD45-PerCPCy5.5. A detailed list of antibodies and the dilutions used is provided in Table S1. FACS data were analyzed using the FlowJo software (TreeStar).

Immunohistochemistry

AGM sections were stained as described previously (Thambyrajah et al., 2016a) and mounted using Prolong Gold Antifade medium with DAPI (Life Technologies). Images were taken using a low-light time-lapse microscope (Leica) using the Metamorph imaging software, and images were processed using ImageJ.

ChIP

ChIP experiments for HDAC1 and HDAC2 were performed with 0.5×10^6 HE2 (CDH5^{+}/CD41^{+}) cells per ChIP according to the manufacturer's instructions (HighCell Kit, Diagenode, cat. no. C01010060). Sequencing libraries were prepared with the Diagenode MicroPlex Library Preparation Kit v.2 (Diagenode, cat. no. C05010012). The ChIP samples were sequenced on an Illumina Hi-Seq 2500.

RNA Extraction and Whole-Genome RNA Sequencing

HE cells (CDH5^{+}/CD41^{+}) were isolated from day 2 cultures of *Hdac1*lox/lox, *Hdac2* lox/lox, *Hdac1*Δ/Δ, and *Hdac2*Δ/Δ. RNA was extracted using the QIAGEN RNAeasy Micro Kit. One nanogram of RNA was processed with SureSelect Strand-Specific RNA Library Prep for Illumina (cat. code: G9691A, Agilent) according to the manufacturer's instructions and run on an Illumina HiSeq as a single ends (1×75 bp) run.

RNA Sequencing Data Analysis

Basecall files generated from a HiSeq sequencing run were converted to FASTQ format with Illumina's bcl2fastq (v.2.17.1.14). Lane-wise alignment was performed by bowtie2 (v.2.2.1) to mouse reference genome mm10 with default parameters. Generated SAM files from bowtie2 alignment were converted to BAM files by samtools v.0.1.19. Parameters for samtools SAM to BAM conversion: -q 10 -f 2 -F 268. Resulting lane-wise BAM files from the same sequence library were merged into one BAM file used for downstream analysis. Read counts from BAM files were extracted under the R environment (R v.3.1.0) with the package Rsubread v.1.13.13. Only genes with at least one count per million in all samples were retained for differential expression analysis. The count table was loaded to edgeR v.3.8.5 and differentially expressed genes between groups were identified by exact test function.

ChIP-Seq Data Analysis

Basecall files generated from HiSeq sequencing run were converted to FASTQ format with Illumina's bcl2fastq. Alignment was performed by bowtie2 (v.2.2.1) to mouse reference genome mm10 with default parameters. Generated SAM files from bowtie2 alignment were converted to BAM files by samtools v.0.1.19. Parameters for samtools SAM to BAM conversion: -bS -q 10 -F 260. Peaks were called using the program macs2 (v. 2.1.0.20150420) with the settings -q 0.01. Full pipeline repo available at: https://github.com/mproffitt/BioWorkflow.

ACCESSION NUMBERS

The sequencing read files from the RNA and ChIP sequencing are available through GEO: GSE101683.

SUPPLEMENTAL INFORMATION

Supplemental Information includes Supplemental Experimental Procedures, seven figures, two tables, and five videos and can be found with this article online at https://doi.org/10.1016/j.stemcr.2018.03.011.

AUTHOR CONTRIBUTIONS

R.T. designed the research, performed experiments, analyzed the data, and wrote the manuscript. M.Z.H.F. analyzed RNA sequencing data and ChIP-seq data and wrote the manuscript. M.P. performed analysis on ChIP data. R.P. performed immunohistochemistry. S.M.C. provided essential reagents and expertise. V.K. and G.L. designed the research, analyzed the data, and wrote the manuscript.

ACKNOWLEDGMENTS

The authors thank the Biological Resources Unit, Advanced Imaging and Flow Cytometry, the Molecular Biology Core Facility, and Histology facilities for technical support and Julia Draper, Michael Lie-a-Ling, and Rui Monteiro for critical reading of the manuscript. Research in the authors' laboratory is supported by the Medical Research Council (MR/P000673/1 to V.K. and MR/J009202/1 to S.M.C.), the Biotechnology and Biological Sciences Research Council (BB/I001794/1 to G.L. and V.K. and BB/N002954/1 to S.M.C.), Bloodwise (12037), the European Union's Horizon 2020 (GA6586250), and Cancer Research UK (C5759/A20971).

Received: July 26, 2017
Revised: March 12, 2018
Accepted: March 13, 2018
Published: April 10, 2018

REFERENCES

Bertrand, J.Y., Chi, N.C., Santoso, B., Teng, S., Stainier, D.Y., and Traver, D. (2010). Haematopoietic stem cells derive directly from aortic endothelium during development. Nature *464*, 108–111.

Boisset, J.C., van Cappellen, W., Andrieu-Soler, C., Galjart, N., Dzierzak, E., and Robin, C. (2010). In vivo imaging of haematopoietic cells emerging from the mouse aortic endothelium. Nature *464*, 116–120.

Budi, E.H., Duan, D., and Derynck, R. (2017). Transforming growth factor-beta receptors and smads: regulatory complexity and functional versatility. Trends Cell Biol. *27*, 658–672.

Burns, C.E., Galloway, J.L., Smith, A.C., Keefe, M.D., Cashman, T.J., Paik, E.J., Mayhall, E.A., Amsterdam, A.H., and Zon, L.I. (2009). A genetic screen in zebrafish defines a hierarchical network of pathways required for hematopoietic stem cell emergence. Blood *113*, 5776–5782.

Chen, M.J., Yokomizo, T., Zeigler, B.M., Dzierzak, E., and Speck, N.A. (2009). Runx1 is required for the endothelial to haematopoietic cell transition but not thereafter. Nature *457*, 887–891.

Choi, K., Kennedy, M., Kazarov, A., Papadimitriou, J.C., and Keller, G. (1998). A common precursor for hematopoietic and endothelial cells. Development *125*, 725–732.

Costa, G., Kouskoff, V., and Lacaud, G. (2012). Origin of blood cells and HSC production in the embryo. Trends Immunol. *33*, 215–223.

Cowley, S.M., Iritani, B.M., Mendrysa, S.M., Xu, T., Cheng, P.F., Yada, J., Liggitt, H.D., and Eizenman, R.N. (2005). The mSin3A chromatin-modifying complex is essential for embryogenesis and T-cell development. Mol. Cell. Biol. *25*, 6990–7004.

Cumano, A., Ferraz, J.C., Klaine, M., Di Santo, J.P., and Godin, I. (2001). Intraembryonic, but not yolk sac hematopoietic precursors, isolated before circulation, provide long-term multilineage reconstitution. Immunity *15*, 477–485.

Dannenberg, J.H., David, G., Zhong, S., van der Torre, J., Wong, W.H., and Depinho, R.A. (2005). mSin3A corepressor regulates diverse transcriptional networks governing normal and neoplastic growth and survival. Genes Dev. *19*, 1581–1595.

de Bruijn, M.F., Speck, N.A., Peeters, M.C., and Dzierzak, E. (2000). Definitive hematopoietic stem cells first develop within the major arterial regions of the mouse embryo. EMBO J. *19*, 2465–2474.

Dieterlen-Lievre, F. (1975). On the origin of haemopoietic stem cells in the avian embryo: an experimental approach. J. Embryol. Exp. Morphol. *33*, 607–619.

Dovey, O.M., Foster, C.T., Conte, N., Edwards, S.A., Edwards, J.M., Singh, R., Vassiliou, G., Bradley, A., and Cowley, S.M. (2013). Histone deacetylase 1 and 2 are essential for normal T-cell development and genomic stability in mice. Blood *121*, 1335–1344.

Dovey, O.M., Foster, C.T., and Cowley, S.M. (2010). Histone deacetylase 1 (HDAC1), but not HDAC2, controls embryonic stem cell differentiation. Proc. Natl. Acad. Sci. USA *107*, 8242–8247.

Eilken, H.M., Nishikawa, S., and Schroeder, T. (2009). Continuous single-cell imaging of blood generation from haemogenic endothelium. Nature *457*, 896–900.

Fehling, H.J., Lacaud, G., Kubo, A., Kennedy, M., Robertson, S., Keller, G., and Kouskoff, V. (2003). Tracking mesoderm induction and its specification to the hemangioblast during embryonic stem cell differentiation. Development *130*, 4217–4227.

Frame, J.M., Fegan, K.H., Conway, S.J., McGrath, K.E., and Palis, J. (2016). Definitive hematopoiesis in the yolk sac emerges from wnt-responsive hemogenic endothelium independently of circulation and arterial identity. Stem Cells *34*, 431–444.

Goode, D.K., Obier, N., Vijayabaskar, M.S., Lie, A.L.M., Lilly, A.J., Hannah, R., Lichtinger, M., Batta, K., Florkowska, M., Patel, R., et al. (2016). Dynamic gene regulatory networks drive hematopoietic specification and differentiation. Dev. Cell *36*, 572–587.

Huang, H.T., Kathrein, K.L., Barton, A., Gitlin, Z., Huang, Y.H., Ward, T.P., Hofmann, O., Dibiase, A., Song, A., Tyekucheva, S., et al. (2013). A network of epigenetic regulators guides developmental haematopoiesis in vivo. Nat. Cell Biol. *15*, 1516–1525.

Jamaladdin, S., Kelly, R.D., O'Regan, L., Dovey, O.M., Hodson, G.E., Millard, C.J., Portolano, N., Fry, A.M., Schwabe, J.W., and Cowley, S.M. (2014). Histone deacetylase (HDAC) 1 and 2 are essential for accurate cell division and the pluripotency of embryonic stem cells. Proc. Natl. Acad. Sci. USA *111*, 9840–9845.

Kelly, R.D., and Cowley, S.M. (2013). The physiological roles of histone deacetylase (HDAC) 1 and 2: complex co-stars with multiple leading parts. Biochem. Soc. Trans. *41*, 741–749.

Kissa, K., and Herbomel, P. (2010). Blood stem cells emerge from aortic endothelium by a novel type of cell transition. Nature *464*, 112–115.

Lacaud, G., Gore, L., Kennedy, M., Kouskoff, V., Kingsley, P., Hogan, C., Carlsson, L., Speck, N., Palis, J., and Keller, G. (2002). Runx1 is essential for hematopoietic commitment at the hemangioblast stage of development in vitro. Blood *100*, 458–466.

Lancrin, C., Mazan, M., Stefanska, M., Patel, R., Lichtinger, M., Costa, G., Vargel, O., Wilson, N.K., Moroy, T., Bonifer, C., et al. (2012). GFI1 and GFI1B control the loss of endothelial identity of hemogenic endothelium during hematopoietic commitment. Blood *120*, 314–322.

Lancrin, C., Sroczynska, P., Serrano, A.G., Gandillet, A., Ferreras, C., Kouskoff, V., and Lacaud, G. (2010). Blood cell generation from the hemangioblast. J. Mol. Med. (berl) *88*, 167–172.

Lancrin, C., Sroczynska, P., Stephenson, C., Allen, T., Kouskoff, V., and Lacaud, G. (2009). The haemangioblast generates haematopoietic cells through a haemogenic endothelium stage. Nature *457*, 892–895.

Lebrin, F., Deckers, M., Bertolino, P., and Ten Dijke, P. (2005). TGF-beta receptor function in the endothelium. Cardiovasc. Res. *65*, 599–608.

McGarvey, A.C., Rybtsov, S., Souilhol, C., Tamagno, S., Rice, R., Hills, D., Godwin, D., Rice, D., Tomlinson, S.R., and Medvinsky, A. (2017). A molecular roadmap of the AGM region reveals BMPER as a novel regulator of HSC maturation. J. Exp. Med. *214*, 3731–3751.

Medvinsky, A., and Dzierzak, E. (1996). Definitive hematopoiesis is autonomously initiated by the AGM region. Cell *86*, 897–906.

Medvinsky, A., Rybtsov, S., and Taoudi, S. (2011). Embryonic origin of the adult hematopoietic system: advances and questions. Development *138*, 1017–1031.

Monteiro, R., Pinheiro, P., Joseph, N., Peterkin, T., Koth, J., Repapi, E., Bonkhofer, F., Kirmizitas, A., and Patient, R. (2016). Transforming growth factor beta drives hemogenic endothelium programming and the transition to hematopoietic stem cells. Dev. Cell *38*, 358–370.

Montgomery, R.L., Davis, C.A., Potthoff, M.J., Haberland, M., Fielitz, J., Qi, X., Hill, J.A., Richardson, J.A., and Olson, E.N. (2007). Histone deacetylases 1 and 2 redundantly regulate cardiac morphogenesis, growth, and contractility. Genes Dev. *21*, 1790–1802.

Muller, A.M., Medvinsky, A., Strouboulis, J., Grosveld, F., and Dzierzak, E. (1994). Development of hematopoietic stem cell activity in the mouse embryo. Immunity *1*, 291–301.

North, T.E., de Bruijn, M.F., Stacy, T., Talebian, L., Lind, E., Robin, C., Binder, M., Dzierzak, E., and Speck, N.A. (2002). Runx1 expression marks long-term repopulating hematopoietic stem cells in the midgestation mouse embryo. Immunity *16*, 661–672.

Okuda, T., van Deursen, J., Hiebert, S.W., Grosveld, G., and Downing, J.R. (1996). AML1, the target of multiple chromosomal translocations in human leukemia, is essential for normal fetal liver hematopoiesis. Cell *84*, 321–330.

Ruetz. (2017). Constitutively active SMAD2/3 are broad-scope potentiators of transcription-factor-mediated cellular reprogramming. Cell Stem Cell *21*, 1–15.

Sroczynska, P., Lancrin, C., Kouskoff, V., and Lacaud, G. (2009a). The differential activities of Runx1 promoters define milestones during embryonic hematopoiesis. Blood *114*, 5279–5289.

Sroczynska, P., Lancrin, C., Pearson, S., Kouskoff, V., and Lacaud, G. (2009b). In vitro differentiation of mouse embryonic stem cells as a model of early hematopoietic development. Methods Mol. Biol. *538*, 317–334.

Stefanska, M., Batta, K., Patel, R., Florkowska, M., Kouskoff, V., and Lacaud, G. (2017). Primitive erythrocytes are generated from hemogenic endothelial cells. Sci. Rep. *7*, 6401.

Taoudi, S., Gonneau, C., Moore, K., Sheridan, J.M., Blackburn, C.C., Taylor, E., and Medvinsky, A. (2008). Extensive hematopoietic stem cell generation in the AGM region via maturation of VE-cadherin+CD45+ pre-definitive HSCs. Cell Stem Cell *3*, 99–108.

Taoudi, S., and Medvinsky, A. (2007). Functional identification of the hematopoietic stem cell niche in the ventral domain of the embryonic dorsal aorta. Proc. Natl. Acad. Sci. USA *104*, 9399–9403.

Thambyrajah, R., Mazan, M., Patel, R., Moignard, V., Stefanska, M., Marinopoulou, E., Li, Y., Lancrin, C., Clapes, T., Moroy, T., et al. (2016a). GFI1 proteins orchestrate the emergence of haematopoietic stem cells through recruitment of LSD1. Nat. Cell Biol. *18*, 21–32.

Thambyrajah, R., Patel, R., Mazan, M., Lie, A.L.M., Lilly, A., Eliades, A., Menegatti, S., Garcia-Alegria, E., Florkowska, M., Batta, K., et al. (2016b). New insights into the regulation by RUNX1 and GFI1(s) proteins of the endothelial to hematopoietic transition generating primordial hematopoietic cells. Cell Cycle *15*, 2108–2114.

Thiery, J.P., Acloque, H., Huang, R.Y., and Nieto, M.A. (2009). Epithelial-mesenchymal transitions in development and disease. Cell *139*, 871–890.

Vargel, O., Zhang, Y., Kosim, K., Ganter, K., Foehr, S., Mardenborough, Y., Shvartsman, M., Enright, A.J., Krijgsveld, J., and Lancrin, C. (2016). Activation of the TGFbeta pathway impairs endothelial to haematopoietic transition. Sci. Rep. *6*, 21518.

Wilting, R.H., Yanover, E., Heideman, M.R., Jacobs, H., Horner, J., van der Torre, J., DePinho, R.A., and Dannenberg, J.H. (2010). Overlapping functions of Hdac1 and Hdac2 in cell cycle regulation and haematopoiesis. EMBO J. *29*, 2586–2597.

Zhou, F., Li, X., Wang, W., Zhu, P., Zhou, J., He, W., Ding, M., Xiong, F., Zheng, X., Li, Z., et al. (2016). Tracing haematopoietic stem cell formation at single-cell resolution. Nature *533*, 487–492.

Zovein, A.C., Hofmann, J.J., Lynch, M., French, W.J., Turlo, K.A., Yang, Y., Becker, M.S., Zanetta, L., Dejana, E., Gasson, J.C., et al. (2008). Fate tracing reveals the endothelial origin of hematopoietic stem cells. Cell Stem Cell *3*, 625–636.

Stem Cell Reports, Volume *10*

Supplemental Information

HDAC1 and HDAC2 Modulate TGF-β Signaling during Endothelial-to-Hematopoietic Transition

Roshana Thambyrajah, Muhammad Z.H. Fadlullah, Martin Proffitt, Rahima Patel, Shaun M. Cowley, Valerie Kouskoff, and Georges Lacaud

Inventory of Supplemental Figures

Supplemental Figure S1: *Hdac1* or *Hdac2* deletion impairs EHT (related to Figure 1 and Figure 2)

Supplemental Figure S2: One allele of *Hdac2* is sufficient for EHT at reduced levels (related to Figure 2 and Figure 3)

Supplemental Figure S3: *Hdac1/2* double knock out diminishes hematopoiesis (related to Figure 3)

Supplemental Figure S4: Apoptosis is increased in *Hdac1/2* (related to Figure 3)

Supplemental Figure S5: Elevated *Caspase3/7* detection in *Hdac1/2* double knock out cultures (related to Figure 3)

Supplemental Figure S6: HDAC1 and HDAC2 ChIP-seq peak distribution (related to Figure 5)

Supplemental Figure S7: Gene expression level of tgfβ, cell cycle and apoptosis related genes (related to Figure 5)

Movie 1 and Movie 2: *Hdac1/2* wild type cultures show low numbers of GFP positive cells (related to Figure 3)

Movie 3- 5: *Hdac1/2* double knock out cells undergo improved EHT with SB43 (related to Figure 6)

A S1: Hdac1 or Hdac2 deletion impairs EHT (related to Figure 1 and Figure 2)

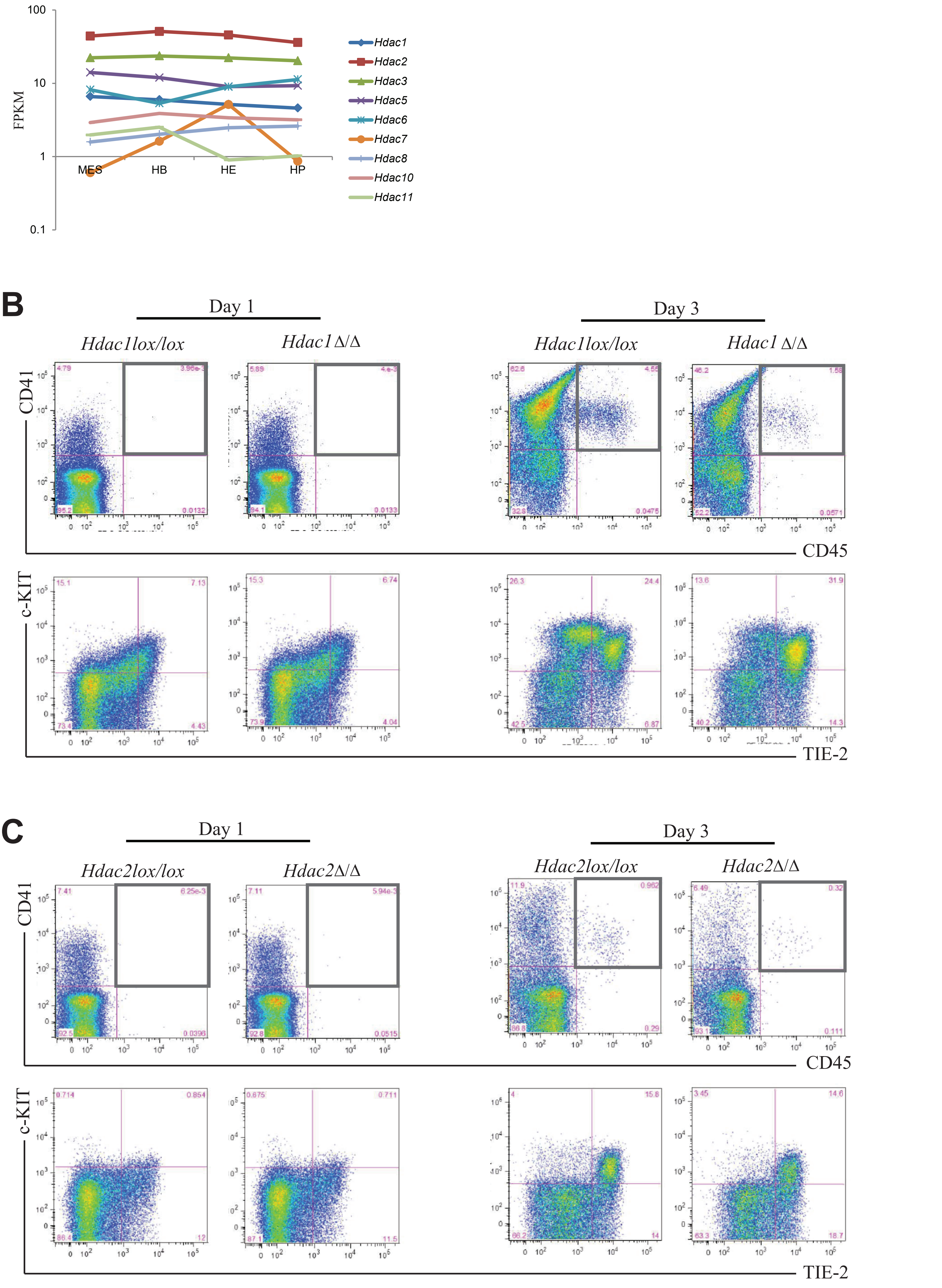

S2 One allele of *Hdac2* is sufficient for reduced EHT (related to Figure 1 and Figure 2)

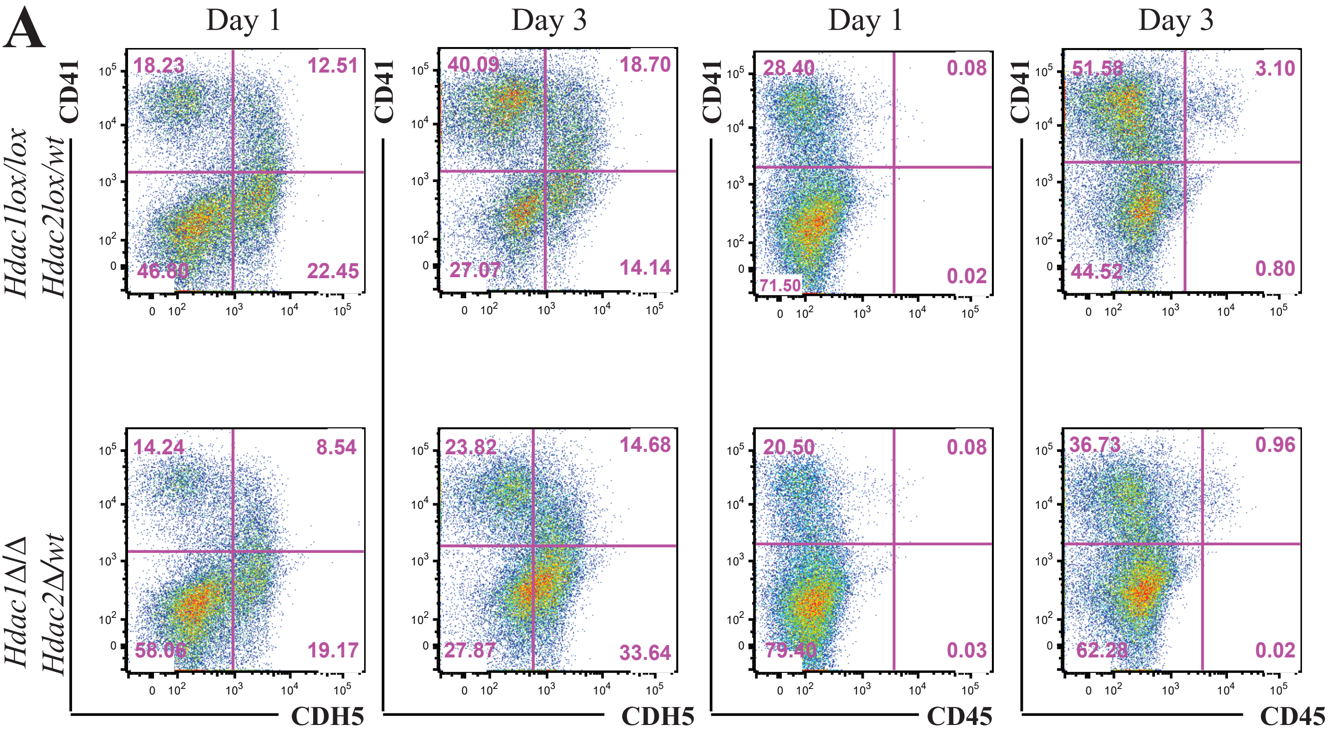

S3: *Hdac1/2* double knock out diminshes hematopoiesis (related to Figure 3)

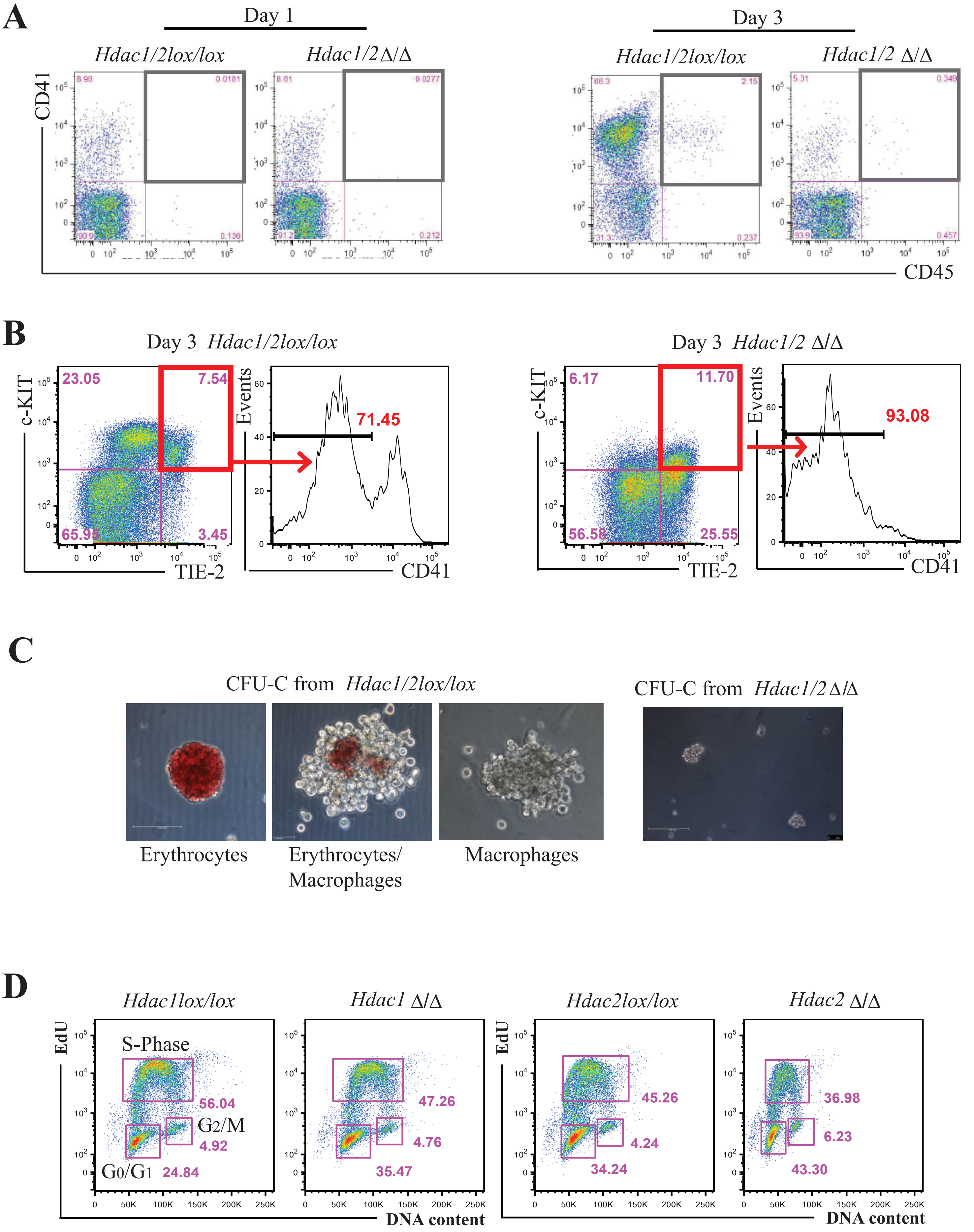

S4 Apoptosis is increased in *Hdac1/2* double KO HE (related to Figure 3)

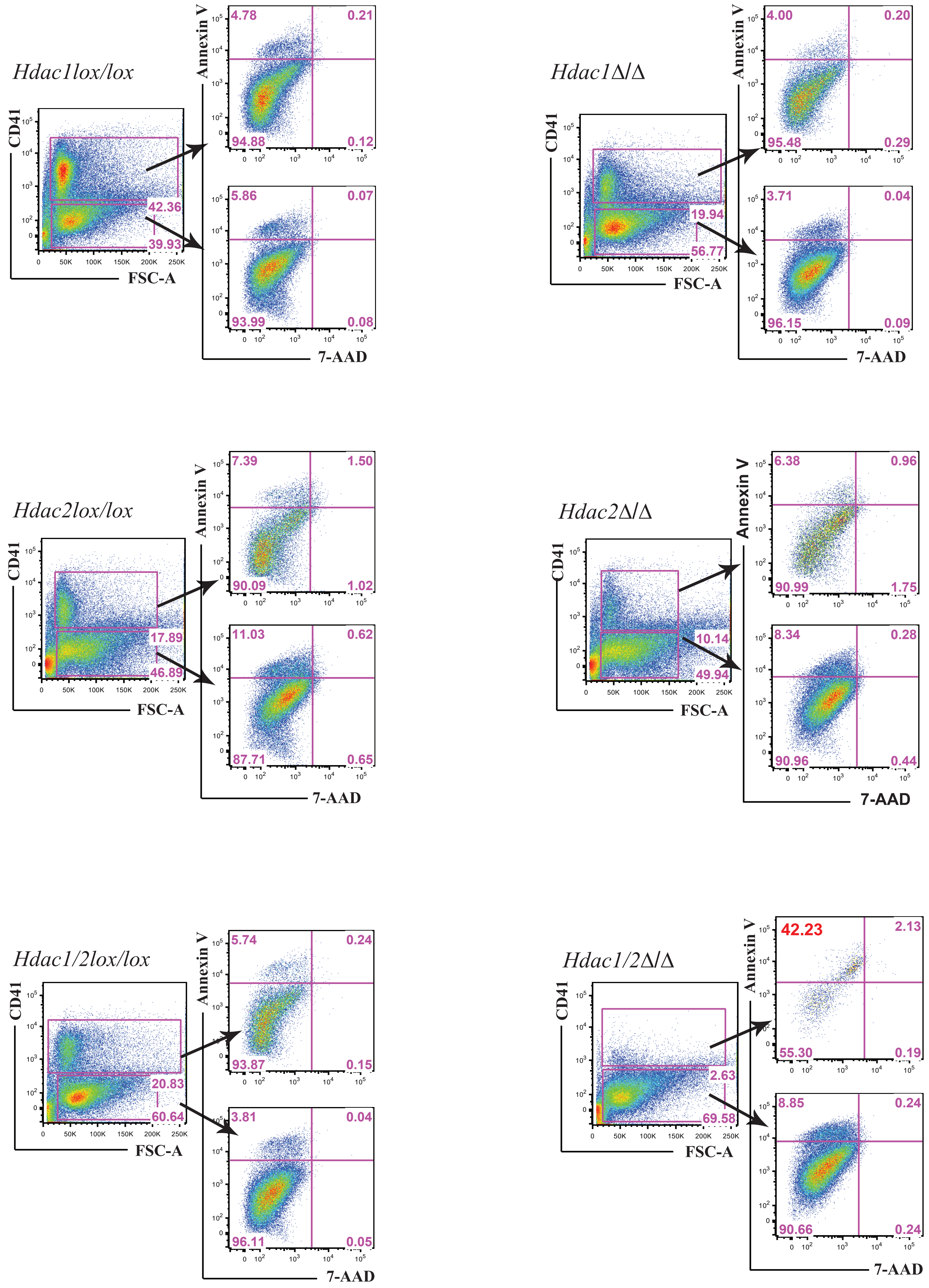

S5 Elevated *Caspase3/7* detection in *Hdac1/2* double KO cultures (related to Figure 3)

A

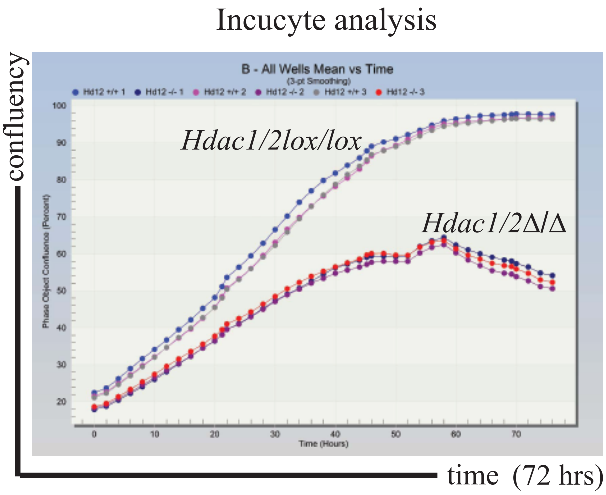

B

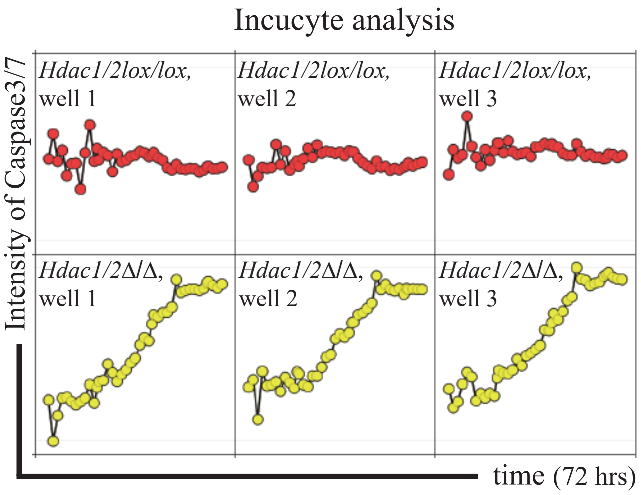

S6: *Hdac1* and *Hdac2* peak distribution (related to Figure 5)

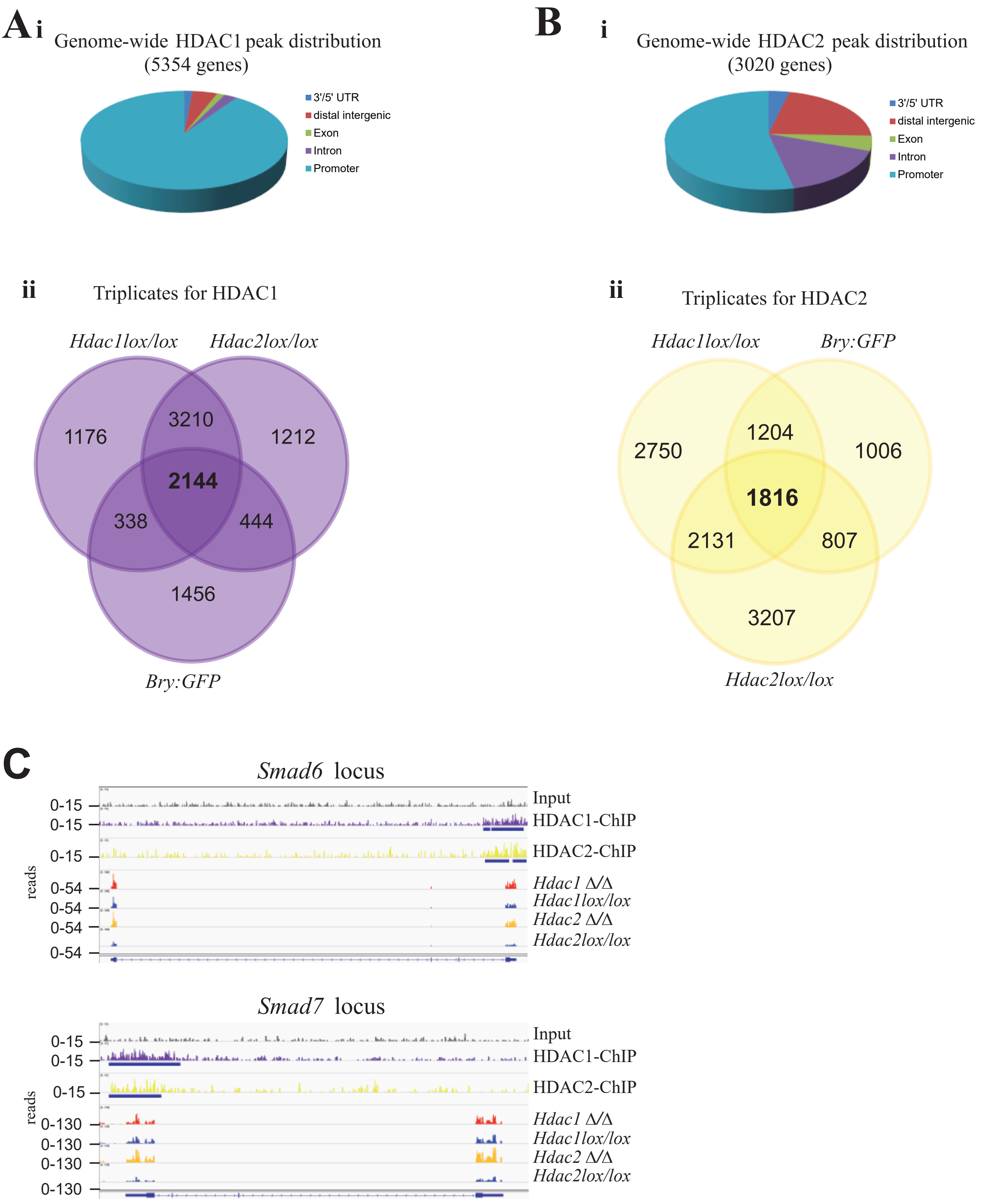

S7 Gene expression level of tgfβ, cell cycle and apoptosis related genes (related to Figure 5)

A Heat map of cell cycle genes

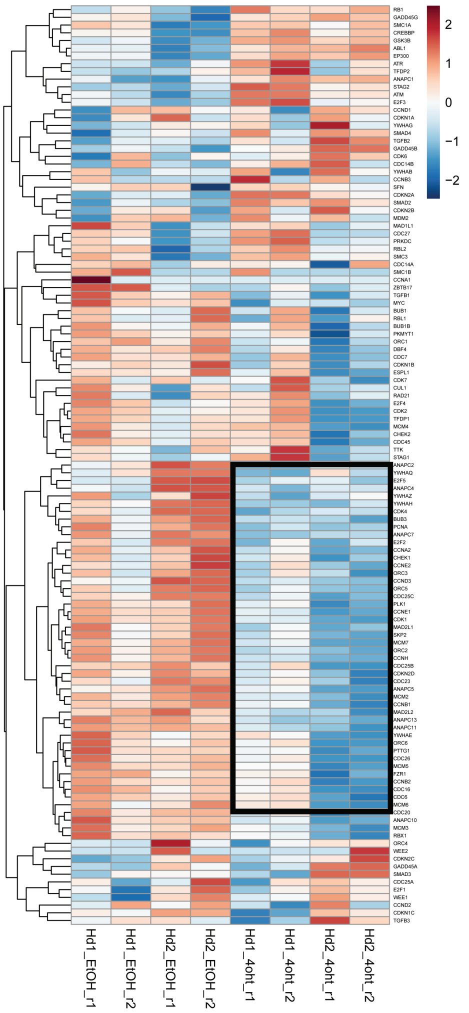

B Heat map of apoptosis genes

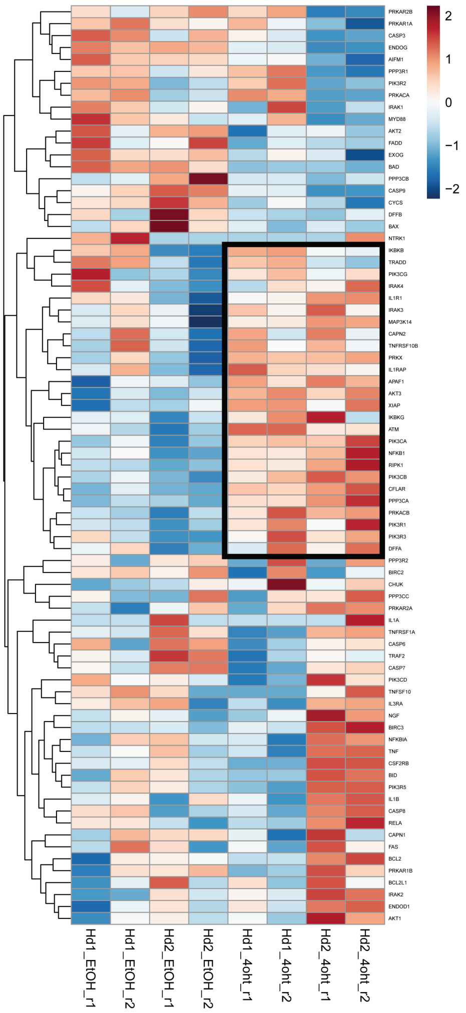

C Heat map of tgfb familiy members

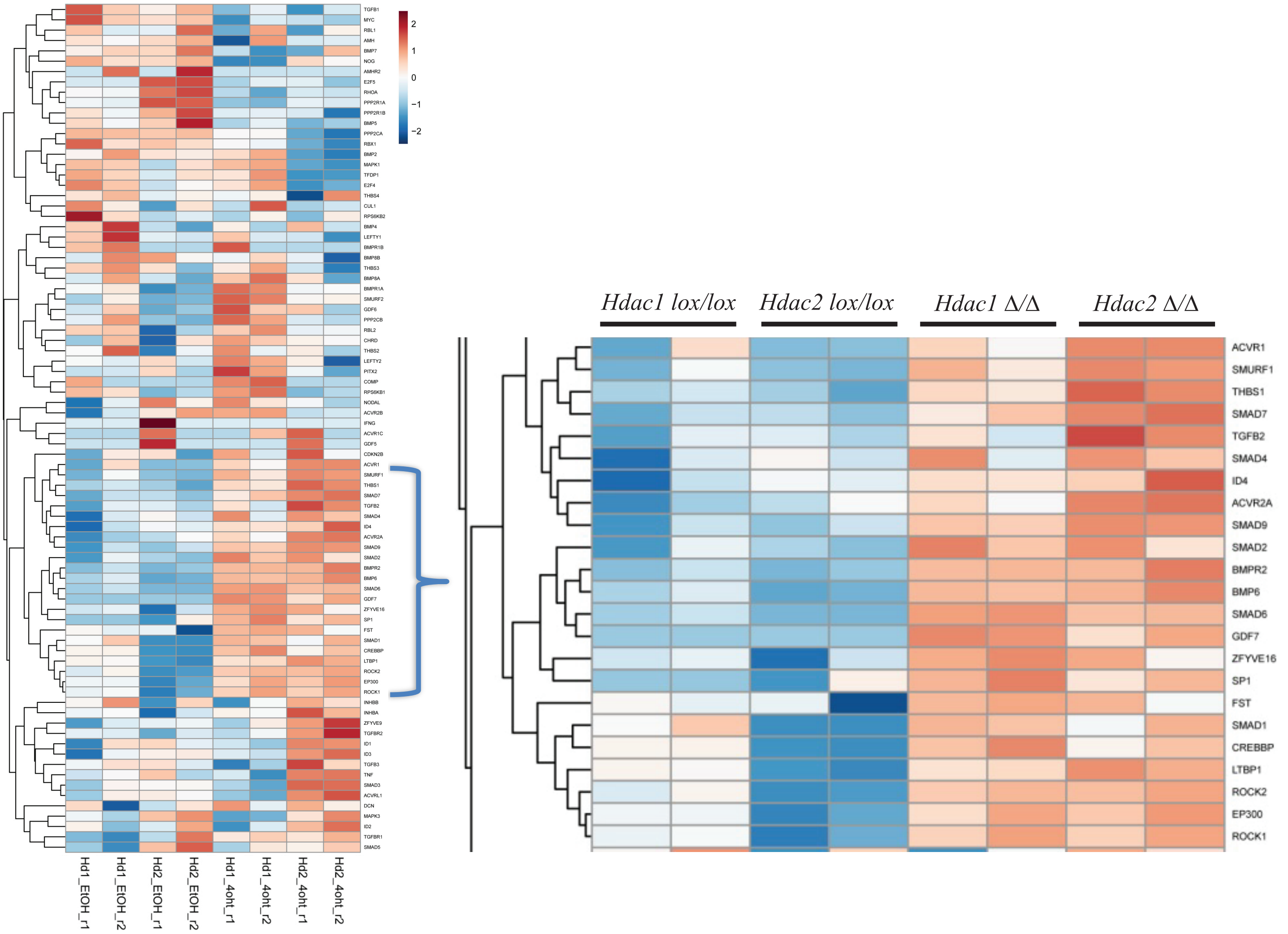

Supplemental Figures

Supplemental Figure S1. HDAC1 or HDAC2 deletion reduces generation of CD45⁺ cells (A) RNA–sequencing read counts (FPKM= Reads Per Kilobase of transcript per Million mapped reads) for members of the HDAC family in the specified populations. MES= mesoderm, HB= hemangioblast, HE and HP= hematopoietic progenitors (from Goode et al, 2016). (B and C) FACS analysis of *Hdac1loxlox* and *Hdac1Δ/Δ,* or *Hdac2loxlox* and *Hdac2Δ/Δ* cultures at day 1 and day 3. Cells were stained for the endothelial marker CDH5 or TIE2, and the hematopoietic markers c-KIT, CD41 and CD45. Gray boxes indicate CD45 expressing cells shown in Figures 2C and 2F.

Supplemental Figure S2 One allele of *Hdac2* is sufficient for hematopoiesis (A) FACS assessment of day 1 and day 3 *Hdac1loxlox*;*Hdac2lox/wt* and *Hdac1Δ/Δ;Hdac2Δ/wt* cultures for endothelial (CDH5) and hematopoietic (CD41 and CD45) markers.

Supplemental Figure S3 Hematopoietic potential of *Hdac1/2* deleted cells is severely reduced (A) FACS assessment of *Hdac1/2loxlox* and *Hdac1/2Δ/Δ* day 1 and day 3 cultures. Cells were stained for the endothelial marker CDH5 or TIE-2, and the hematopoietic markers c-KIT, CD41 and CD45. Gray boxes indicate CD45 expressing cells shown in Figures 2G. (B) FACS for the endothelial marker TIE-2 and the hematopoietic markers c-KIT and CD41 at day 3 of Li-Blast culture. The red box highlights the cells selected for CD41 expression analysis. (C) Representative images of CFU-C from day 3 *Hdac1/2*lox/lox and *Hdac1/2*Δ/Δ Scale bar: 1mm. (D) FACS plots of EdU cell cycle analysis of day 2 *Hdac1lox/lox*, *Hdac1Δ/Δ*, *Hdac2lox/lox* and *Hdac2Δ/Δ* day 2 Li-Blast cultures. Representative plots of two independent experiments.

Supplemental Figure S4 Apoptosis is significantly increased in *Hdac1/2Δ/Δ* FACS assessment for apoptosis of *Hdac1lox/lox*, *Hdac2*lox/lox and *Hdac1/2*loxlox lines with/out 4OHT treatment. Day 3 cultures were stained for the hematopoietic marker CD41, the cell viability marker 7-AAD and the apoptosis marker Annexin 5. Representative plots of two experiments.

Supplemental Figure S5 Caspase3/7 intensity is significantly increased in *Hdac1/2 Δ/Δ* Extracts from Incucyte time-lapse imaging of the *Hdac1/2loxlox* lines with/out 4OHT, starting from Day1 for 72 hours. (A) Graph showing the confluency of the cultures over 72 hour period. (B) Apoptosis analysis with GFP labelled *Caspase3/7* reagent. GFP is only emitted once it binds to cleaved *Caspase3/7.* The experiment was performed in triplicates.

Supplemental Figure S6 Summary of the ChIP-Seq data for *Hdac1* and *Hdac2* (A) Summary of the ChIP-Seq binding profile for *Hdac1*. (Ai) peak distribution in genomic locations (Aii) Venn diagram of the three ChIP-Seq replicates (B) Summary of the ChIP-Seq data for *Hdac2*. (Bi) peak distribution in genomic locations (Bii) Venn diagram of the three ChIP-Seq replicates. (C) Representative extracts from the Genome Browser (IGV). Two loci, Smad6 and Smad7, are shown with visualisation of the binding sites of HDAC1 and HDAC2 and the RNA-sequencing results of *Hdac1loxlox* and *Hdac1Δ/Δ,* or *Hdac2loxlox* and *Hdac2Δ/Δ*.

Supplemental Figure S7 Heat map of cell cycle, apoptosis and *Tgfβ* related genes Gene lists of interest were retrieved from the GSEA data bank. The expression level of the RNA-Seq data from *Hdac1lox/lox*, *Hdac2lox/lox*, *Hdac1Δ/Δ* and *Hdac2Δ/Δ* was plotted as a heat map for the gene families of interest. (A) Heat map of cell cycle related genes. (B) Heat map of apoptosis related genes. (C) Heat map of *Tgfβ* family members.

Movie 1 and movie 2 ***Hdac1/2*** **wild type cultures show low numbers of GFP positive cells** Flk1 positive cells from day 3 EBs of the *Hdac1/2loxlox* line were sorted and treated with ethanol (movie1 *Hdac1/2* wild type) or with 4-OHT in Li-Blast culture (*Hdac1/2* double knock out). One day later, GFP labelled Caspase3/7 antibody was added to the culture and imaged every 2 hours.

Movie 3- 5 ***Hdac1/2*** **double knock out cells undergo improved EHT with SB43** Flk1 positive cells from day 3 EBs of the *Hdac1/2loxlox* line were sorted and treated with ethanol (movie3 *Hdac1/2* wild type), with 4-OHT (*Hdac1/2* double knock out), or 4-OHT and SB43 combined (*Hdac1/2* double knock out with SB43). Starting from a day later, the cells were imaged every hour.

Supplementary Procedures

ES cell culture and in vitro differentiation

For differentiation, ES cells are seeded on irradiated murine embryonic fibroblast feeder cells until 70-80% confluency (2-3 days). After this initial step, the ES cells are primed for differentiation by passaging them twice onto 0.1% gelatin coated plates. During the last passage, the media is switched from DMEM to an IMDM based media. The ES cells are then allowed to differentiate as embryoid bodies (EB) by dissociating ES cells and plating them in low attachment Petri dishes in differentiation media (IMDM,15% FCS, 2 mM l-glutamine (Gibco), 200 µg/ml transferrin (Roche), 0.5 mM ascorbic acid (Sigma) and 4.5×10-4 M MTG) at a density of 25,000-30,000 cells/ml. After 3-3.5 days, hemangioblast progenitors are isolated on the basis of FLK1 expression (Fehling et al., 2003) and replated in hematopoiesis promoting conditions (Li-Blast). For FACS, time-lapse imaging or re-plating into CFU-C assays, these cultures are initiated with 6.5x 10^4 cells per well in a 6-well plate.

CFU-C assay

In brief, 7-10 days old HE/IAHC cultures or day 3 Li-Blast cultures were trypsinized into single cell suspension. For HE/IAHC cultures, 1/10 of the whole culture was plated into three dishes of CFU-C assay, and hematopoietic colonies scored after 7-10 days. For ES cell based Li-Blast cultures, the number of cells in culture was determined, and 25,000 cells were plated in triplicates into CFU-C assay. The hematopoietic colonies were scored 7-10 days later. The CFU-C mix contains the following cytokines and media: Methylcellulose (1.1% final), 1.5 mL of PDS (15% final), PFHM-II (10% final), L -glutamine (2 mM), transferrin (180 mg/mL), MTG (4.5×10^{-4} M), ascorbic acid stock (50 ng/mL), Kit Ligand (KL) (100 ng/mL), IL-3 (1 ng/mL), G-CSF (30 ng/mL), IL-11 (5 ng/mL), erythropoietin (4 U/mL), IL-6 (10 ng/mL), TPO (5 ng/mL), M-CSF (10 ng/mL).

ImmunoHistochemistry

E10.5 embryos were fixed in 4% Paraformaldehyde for two hours, before they were soaked in 30% sucrose and mounted in OCT compound. 10µm sections were prepared using a cryostat. The sections were streptavidin/biotin blocked if a biotin antibody was used followed by serum blocking (PBS with 10% FCS, 0.05% Tween20 and of 10% goat serum (DAKO) for 1 hour before the sections were incubated with primary antibodies at 4°C overnight in blocking buffer. Sections were washed three times in PBST for 15 minutes each and then incubated with fluorochrome-conjugated secondary antibody at room temperature for 1 hour. Sections were further washed three times in PBS and mounted using Prolong Gold anti-fade medium with DAPI (Life

Technologies). Images were taken using a low-light time lapse microscope (Leica) using the Metamorph imaging software and processed using ImageJ.

***In vitro* culture of AGM cells on OP-9 stromal cells**

AGMs of E10-E10.5 embryos were dissected in PBS with 7% fetal calf serum (FCS) and penicillin/streptomycin (100 U/mL). Single cell suspensions were generated by incubating the tissues for 20-30 minutes in 500 µl of 1mg/ml of Collagenase/Dispase (Roche cat. 10269638001) before mechanical dissociation with a syringe and needle. The resulting single cell suspension was used for antibody staining and FACS sorting. Single AGM cells were sorted into individual irradiated (30 Grays) OP-9 coated wells, and cultured for 7 days in re-aggregation medium consisting of IMDM (Invitrogen), 20% fetal calf serum, L-glutamine (4 mM), penicillin/streptomycin (50 units/ml), mercaptoethanol (0.1 mM), IL-3 (100 ng/ml), SCF (100 ng/ml) and Flt3L (100 ng/ml). All growth factors were purchased from Peprotech. Tissues were maintained in 5% CO2 at 37°C in a humidified incubator, and hematopoietic colonies were scored 7-10 days later.

Genotyping PCR

Small pieces of embryonic tissue or yolk sac were dissected off the embryo and placed in PCR tube containing 30ml of PBS. The tissue pieces were boiled for 8 minutes at 98°C for denaturation. The tissues were digested with Proteinase K (50 mg/ml) for 30 minutes at 55°C, and the enzyme deactivated by boiling the samples for a further 10 minutes at 95°C. 1ml of the samples was used as a template for the PCR.

Western Blot

Cells were washed with ice-cold PBS and lysed using NP-40 lysis buffer (150 mM NaCl, 50mM Tris-HCl pH 8, 1% NP-40 and Proteases Inhibitor Cocktail). After 30 min incubation at room temperature, the lysate was centrifuged at 16,000g for 10 min. The supernatants of whole protein extracts were aliquoted and stored at – 80°C. Protein concentrations were determined using the Bradford Protein Assay (Bio-Rad). Immediately before loading, aliquots of 30 µg of protein lysates were incubated for 10 min at 70°C in gel-loading buffer (NuPage LDS sample buffer and NuPage reducing agent; Invitrogen). The electrophoresis were performed with Sodium Dodecyl Sulfate Polyacrylamide Gel Electrophoresis (SDS PAGE) with NuPAGE 4-12% Bis-Tris gels in XCell SureLock™ Mini-Cell Electrophoresis System containing MES buffer (Invitrogen). The proteins were transferred onto a nitrocellulose membrane using the iBlot® Gel Transfer Stacks Nitrocellulose (Invitrogen) for 13 min. Immuno blottings were performed with iBind™ Western Device (Thermo Fischer Scientific) according to the manufacturer's protocol. 1:200 dilution of the HDAC1 (Diagenode, pAb-053-050), HDAC2 (abcam, ab16032) and β-Actin (Sigma, A5441) were used. The secondary rabbit and goat antibodies were used at a 1:1000 dilution. Finally, the membranes were developed using the Amersham ECLTM Prime Western Blotting Detection Reagent (GE Healthcare), according to the manufacturer's protocol. Films were exposed and developed in a MAS automated developing machine.

Supplemental References

Dovey, O.M., Foster, C.T., and Cowley, S.M. (2010). Histone deacetylase 1 (HDAC1), but not HDAC2, controls embryonic stem cell differentiation. Proc Natl Acad Sci U S A *107*, 8242-8247.

Fehling, H.J., Lacaud, G., Kubo, A., Kennedy, M., Robertson, S., Keller, G., and Kouskoff, V. (2003). Tracking mesoderm induction and its specification to the hemangioblast during embryonic stem cell differentiation. Development *130*, 4217-4227.

Sroczynska, P., Lancrin, C., Pearson, S., Kouskoff, V., and Lacaud, G. (2009). In vitro differentiation of mouse embryonic stem cells as a model of early hematopoietic development. Methods Mol Biol *538*, 317-334.

Thambyrajah, R., Mazan, M., Patel, R., Moignard, V., Stefanska, M., Marinopoulou, E., Li, Y., Lancrin, C., Clapes, T., Moroy, T., *et al.* (2016). GFI1 proteins orchestrate the emergence of haematopoietic stem cells through recruitment of LSD1. Nat Cell Biol *18*, 21-32.
